# Derivatives of the triaminoguanidinium ion, 3. Multiple *N*-functionalization of the triaminoguanidinium ion with isocyanates and isothiocyanates

**DOI:** 10.3762/bjoc.10.234

**Published:** 2014-09-24

**Authors:** Jan Szabo, Kerstin Karger, Nicolas Bucher, Gerhard Maas

**Affiliations:** 1 Institute of Organic Chemistry I, University of Ulm, Albert-Einstein-Allee 11, D-89081 Ulm, Germany, Fax: (+49) 731-50-22803; 2 (new address) TUM CREATE, Singapore 138602, Singapore

**Keywords:** aryl isocyanate, aryl isothiocyanate, carbamoylation, sulfonyl isocyanate, triaminoguanidinium salt, 1*H*-1,2,4-triazole-5(4*H*)-thione

## Abstract

1,2,3-Triaminoguanidinium chloride was combined with benzaldehyde and hydratropic aldehyde to furnish the corresponding tris(imines), which were converted into 1,2,3-tris(benzylamino)guanidinium salts by catalytic hydrogenation in the former, and by borane reduction in the latter case. The resulting alkyl-substituted triaminoguanidinium salts underwent a threefold carbamoylation with aryl isocyanates to furnish 1,2,3-tris(ureido)guanidinium salts, while *p*-toluenesulfonyl isocyanate led only to a mono-ureido guanidinium salt. With aryl isothiocyanates, 3-hydrazino-1*H*-1,2,4-triazole-5(4*H*)-thione derivatives were obtained. Compounds **7a** and **8** show interesting solid-state structures with intra- and intermolecular hydrogen bonds.

## Introduction

The 1,2,3-triaminoguanidinium ion, [C(NHNH_2_)_3_]^+^, appears as an attractive *C*_3_-symmetrical molecular platform, which lends itself to a variety of chemical transformations. Of particular interest should be a threefold chemical functionalization at the three branches of this molecular building block, whereby the original threefold symmetry is maintained. Molecular architectures of this kind could be useful under several aspects, for example in biologically active compounds, as novel ligands in metal complexes, for the construction of dendrimers, and in crystal engineering. Along these lines, several 1,2,3-tris(iminyl)guanidines have been prepared from triaminoguanidinium chloride (**1**, TAG-Cl) and salicylaldehyde, ring-substitued derivatives thereof [2–5] and 3-(hydroxyimino)pentane-2,4-dione [6] to prepare 1,2,3-tris(iminyl)guanidines; as multidentate chelating ligands they form complexes with several metal ions giving rise to various supramolecular coordination architectures in the solid state.

TAG-Cl (**1**) was first prepared from carbon tetrachloride and hydrazine by R. Stollé in the year 1904 [7], but it was not until half a century later that some interest in its chemistry emerged. Derivatization reactions with aldehydes and ketones furnished tris(arylideneamino)- and tris(alkylideneamino)guanidinium salts [7–9]; these were the first examples of a threefold symmetrical functionalization of the triaminoguanidinium ion. On the other hand, reactions of triaminoguanidinium salts with carboxylic acids generated 4-amino-3-hydrazinyl-1,2,4-triazoles [10–11]. Diversely substituted 1,2,4-triazoles were also obtained from reactions with cyanogen bromide [12], CS_2_/NaOH [12] and isothiocyanates [13]. In all these cases, cyclization took place after one or two of the three branches of the triaminoguanidinium ion had been functionalized. Reactions of TAG-Cl (**1**) with 1,3-diketones follow condensation/cyclization pathways resulting in unsymmetrical *N*-heterocyclic products [14–15]. Azidation of TAG-Cl with two equivalents of sodium nitrite in water afforded the highly shock- and friction-sensitive (5-azido-1*H*-tetrazol-1-yl)carbonimidoyl diazide rather than the 1,2,3-triazidoguanidinium salt [16–17].

Direct *N*-alkylation reactions of the triaminoguanidinium ion are not known. Triaminoguanidinium salts are soluble only in water or in hot alcohol/water mixtures, but not in the common organic solvents, which prevents the use of alkylating reagents such as alkyl halides, tosylates and triflates. In our hands, solvent-free heterogenous mixtures of the solid guanidinium salt and a liquid alkylating reagent did not react as desired. Therefore, we developed a two-step alkylation procedure, by which the catalytic hydrogenation of 1,2,3-tris(benzyliminyl)guanidinium chloride, obtained from the condensation of TAG-Cl (**1**) and benzaldehyde, afforded 1,2,3-tris(benzylamino)guanidinium chloride [18]. This salt as well as other ones, obtained by anion exchange reactions, showed interesting solid-state structures, for example with anion-filled channels between layers occupied by the 1,2,3-tris(benzylamino)guanidinium cations [18].

The 1,2,3-tris(alkylamino)guanidinium salts turned out to be sufficiently soluble in several common organic solvents, and so the door is open for further functionalization at the three branches of their cations. In this paper, we report on their reactions with isocyanates and isothiocyanates.

## Results and Discussion

### Synthesis of 1,2,3-tris(alkylamino)guanidinium salts **3** and **5**

**Scheme 1:**
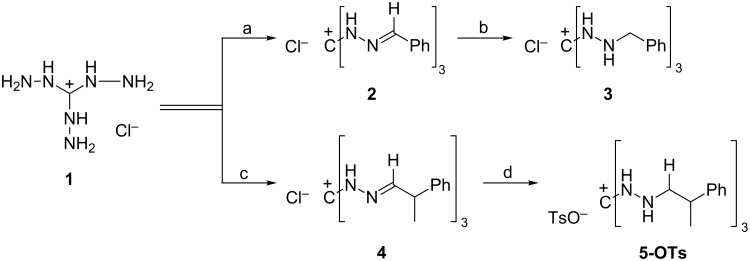
Conditions: a) benzaldehyde, ethanol/water, reflux, 1 h, 96% yield; b) H_2_, Pd/C (10%), MeOH, rt, 48 h, 78%; c) *rac*-Ph−CH(CH_3_)−CHO, water, ultrasound, 2 h, 72%; d) 1. dimethylaminoborane, *p*-toluenesulfonic acid, CH_2_Cl_2_, MeOH, rt, 1.5 h; 2. Na_2_CO_3_ aq, rt, 1 h, 84%.

For the reasons explained in the Introduction, the synthesis of 1,2,3-tris(alkylamino)guanidinium salts from TAG-Cl (**1**) requires a two-step procedure. We have already reported the conversion of **1** into 1,2,3-tris(benzylamino)guanidinium chloride (**3**) via 1,2,3-tris(benzyliminyl)guanidinium chloride (**2**) [18] (Scheme 1). Synthesis of salt **5** by the same strategy requires modifications in both steps, however. It has been stated without further information [8] that salt **1** reacts “anomalously” with hydratropic aldehyde. From our own experiments, we conclude that the earlier failure resulted from a solvent problem, as **1** dissolves well in water, but hydratropic aldehyde does not. We found that the condensation reaction leading to tris(imine) **4** occurs in good yield (72%) under sonication of the reaction mixture with ultrasound.

Concerning the conversion of salt **4** into a 1,2,3-tris(2-phenylpropylamino)guanidinium salt **5**, all attempts to reduce **4** by catalytic hydrogenation failed, and classical reducing agents such as sodium borohydride and lithium aluminum hydride showed no success either. According to a procedure by Casarini et al. [19] compound **5** could finally be obtained. This procedure uses dimethylaminoborane/*p*-toluenesulfonic acid, where the acid serves as an activator to release free borane. With a large excess of *p*-toluenesulfonic acid an anion exchange also occurred, leading to the corresponding tosylate **5-OTs** instead of the chloride. Hydrochloric acid can also be used as an activator. However, the corresponding chloride salt **5-Cl** was not obtained in an analytically pure form, which is why we continued to work with the tosylate. Since we used racemic hydratropic aldehyde, two pairs of enantiomers (*RRR/SSS*, *RRS/SSR*) could be expected. The ^1^H and ^13^C NMR spectra suggest that only the *C*_3_-symmetric *RRR*/*SSS* pair is formed, as only one set of signals, showing just the expected number of signals for a *C*_3_-symmetric cation, is observed.

### Reaction of salts **3** and **5** with isocyanates

**Scheme 2:**
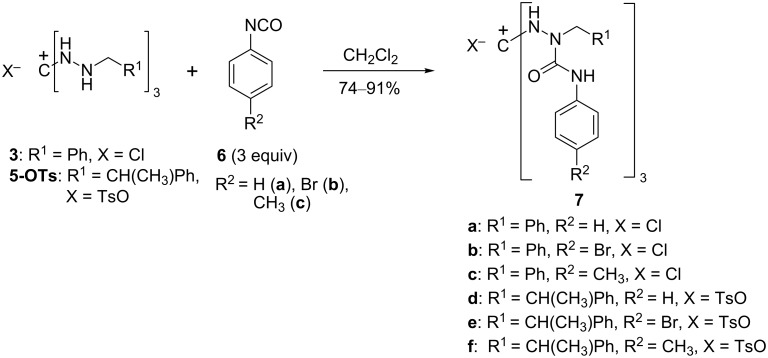
Carbamoylation of 1,2,3-tris(benzylamino)guanidinium salts **3** and **5-OTs**.

When 1,2,3-tris(benzylamino)guanidinium salts **3** and **5-OTs** were combined with three mol equivalents of aryl isocyanates **6a**–**c**, triply carbamoylated salts **7a**–**f** were obtained in good to high yields (Scheme 2). As was expected on the basis of the Hammett σ_p_ values [20], *p*-tolyl isocyanate (**6c**) reacted much more slowly than **6a**,**b**.

**Figure 1:**
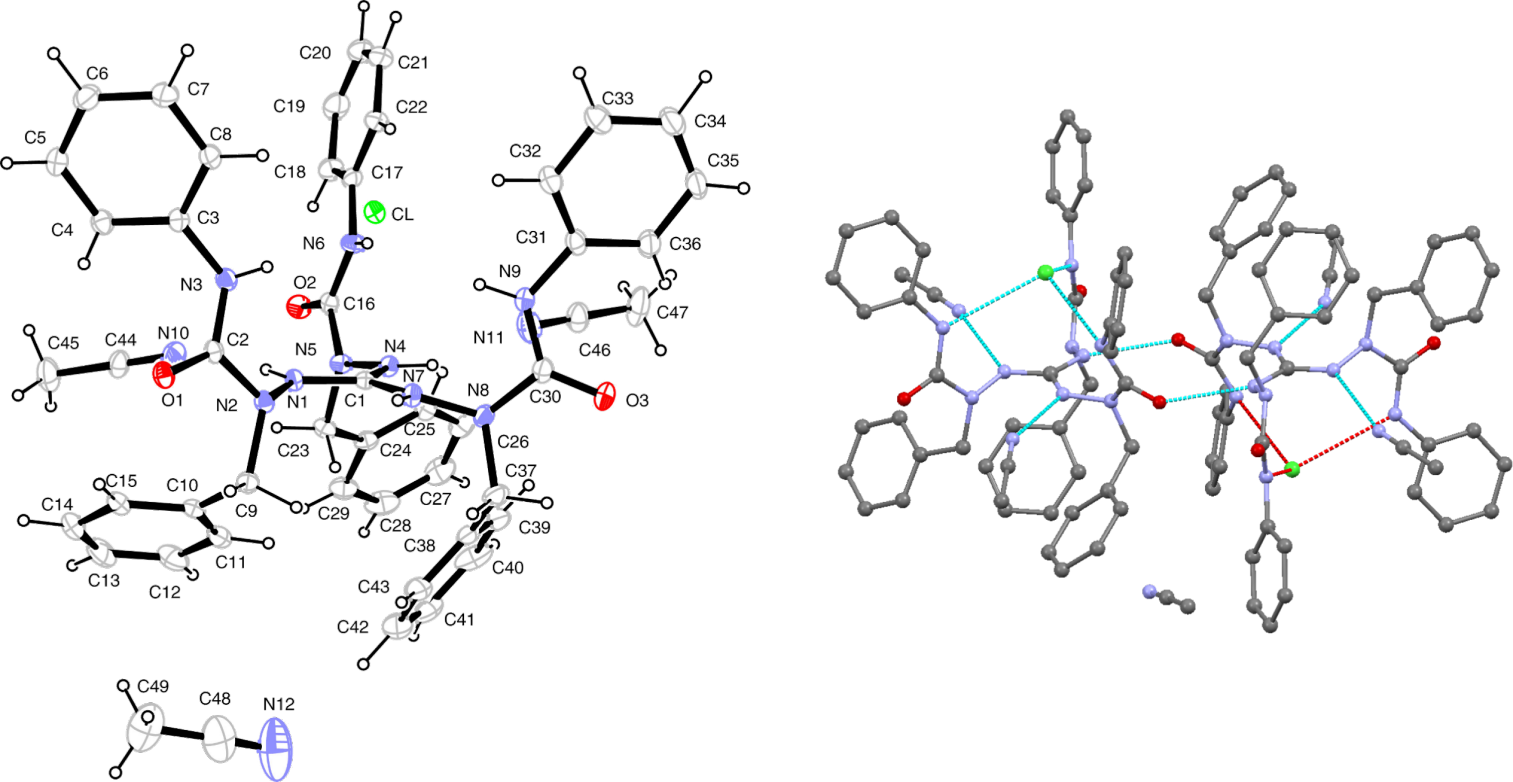
Solid-state structure of **7a**·3CH_3_CN. Left: Molecular structure with numbering of atoms. Right: N–H···Cl, N–H···N(acetonitrile) and N–H···O hydrogen bonds. Thermal displacement ellipsoids are drawn at the 30% probability level. Selected bond lengths (Å): C1–N1 1.330(3), C1–N4 1.333(3), C1–N7 1.332(3), N1–N2 1.397(3), N4–N5 1.388(3), N7–N8 1.401(3). Selected bond angles (°): N1–C1–N4 119.0(2), N1–C1–N7 120.7(2), N4–C1–N7 120.3(2). For torsion angles and hydrogen bonds, see Table 1 and Table S4 (Supporting Information File 1).

The molecular structure of **7a·**3CH_3_CN in the solid state was established by a single crystal X-ray diffraction analysis and is shown in Figure 1 (vide infra for a discussion of structural details). It is interesting to note that the chloride anion is trapped inside a cavity of the cation, being fixed there by three N–H···Cl hydrogen bonds; this suggests that the cation can also serve as a receptor for other anions of appropriate size and shape.

**Scheme 3:**
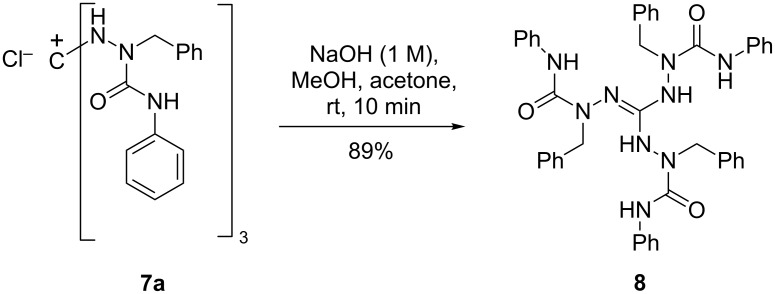
Deprotonation of **7a** to yield the neutral guanidine derivative **8**.

**Figure 2:**
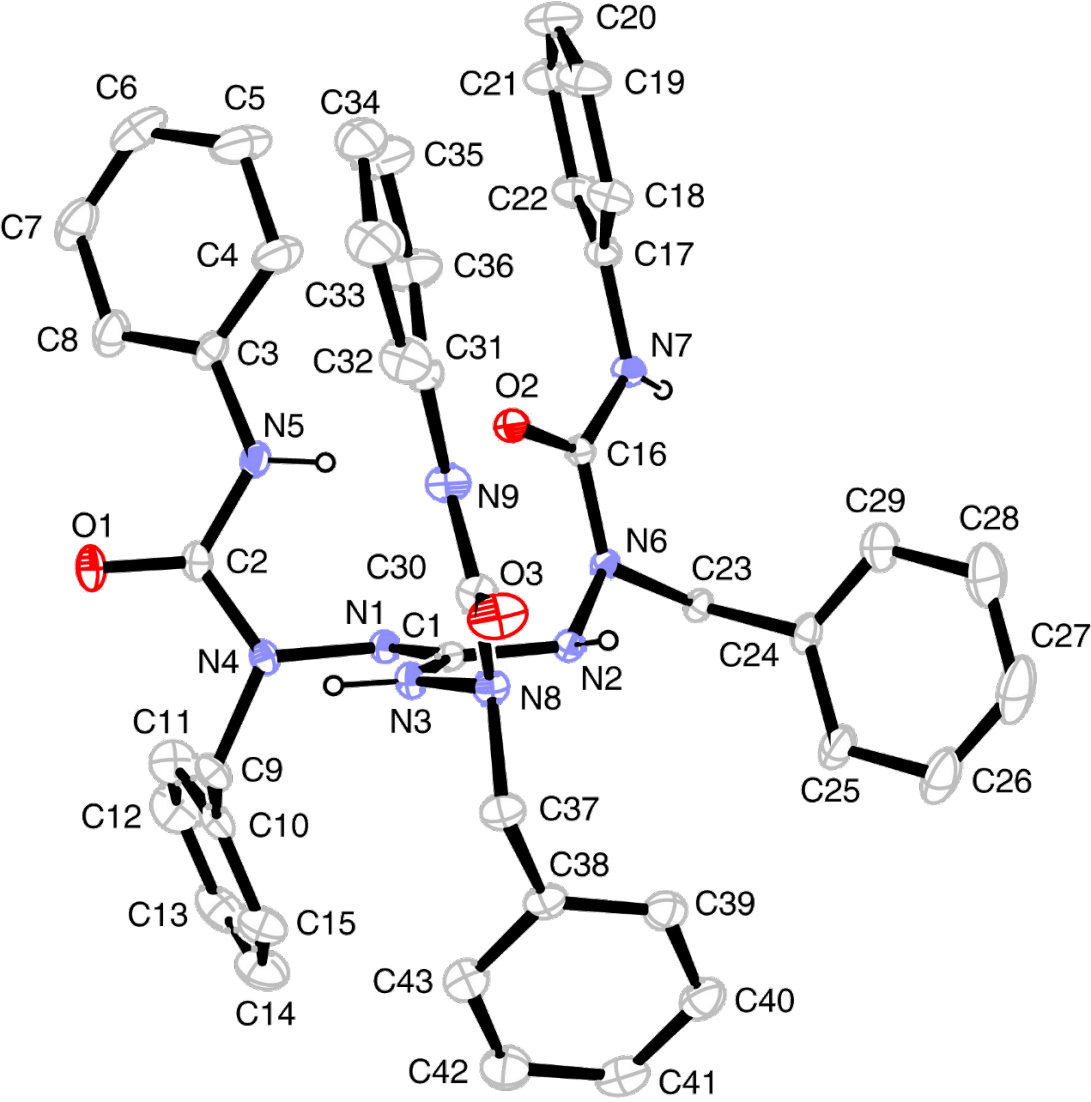
Solid-state structure of **8**. Thermal displacement ellipsoids are drawn at the 20% probability level. Only NH hydrogen atoms are shown, CH hydrogen atoms are omitted for the sake of clarity. Selected bond lengths (Å): C1–N1 1.305(2), C1–N2 1.382(2), C1–N3 1.351(2), N1–N4 1.433(2), N2–N6 1.407(2), N3–N8 1.407(2). Selected bond angles (°): N1–C1–N2 118.8(1), N1–C1–N3 125.0(1), N2–C1–N3 116.2(1). For hydrogen bonds and torsion angles, see Table 1 and Table S4 (Supporting Information File 1).

The triaminoguanidinium salt **7a** was easily deprotonated to afford the corresponding neutral tris(ureido)guanidine **8** (Scheme 3), the structure of which was confirmed by single-crystal X-ray analysis (Figure 2, vide infra for a structural comparison with **7a**).

**Scheme 4:**
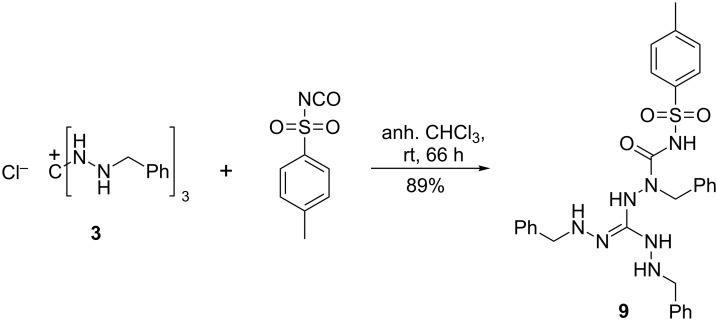
Sulfonylcarbamoylation of salt **3**.

When (*p*-toluenesulfonyl) isocyanate instead of phenyl isocyanate was allowed to react with 1,2,3-tris(benzylamino)guanidinium chloride (**3**), a mono-ureido 1,2,3-triaminoguanidine **9** was obtained, no matter whether a 1:1 or a 3:1 stoichiometry of the reactants was applied (Scheme 4). Notably, not the expected guanidine hydrochloride but the neutral guanidine **9** was obtained. Obviously, the basicity of guanidine **9** is much lower than, for example, the parent compound, 1,2,3-triaminoguanidine. Attempts to convert **9** into the corresponding triaminoguanidinium salt with strong acids failed.

### Structural and NMR data of guanidinium salts **7** and guanidine derivative **8**

The solid-state structures of guanidinium salt **7a** and the neutral guanidine derivative **8** display a common structural motif: the guanidine core (CN_3_)^+^ constitutes a platform with the three polar arylaminocarbonyl in the upper hemisphere and the unpolar benzyl groups in the lower (Figure 1 and Figure 2). On the other hand, an inspection of relevant bond parameters (Figure 1 and Figure 2) and torsion angles (Table S4 in Supporting Information File 1) reveals significant differences between the two structures. The C–N bond lengths of the guanidinium unit in **7a** are equal (1.330(3)–1.333(3) Å) and indicate the full charge delocalization and partial double bond character of these bonds. In the neutral guanidine **8** this symmetry is lost; one finds a short (1.305 Å) and two longer (1.351, 1.382 Å) carbon–nitrogen bonds. The marked difference of the two longer bonds is related to different torsion angles around the C1–N2 and C1–N3 bonds as well as different types of hydrogen bonds – an intramolecular N2–H···N8 and an intermolecular N3–H···O2(=C) hydrogen bond (Table 1).

The conformational differences in the upper, polar hemisphere of **7a** and **8** are also evident. In **7a**, the three carbonyl groups point to the exterior and the three N–H bonds to the interior of the cavity formed by the three urea branches. In this way, the chloride anion is trapped in the inside of the cavity and is held in position by three N-H···Cl hydrogen bonds (*d*(N···Cl) = 3.102–3.275 Å). On the contrary, in the neutral guanidine derivative **8** two N–H bonds and the carbonyl group of the third branch occupy the interior of the cavity, giving rise to two intramolecular hydrogen bonds (N5–H···O2 and N9–H···O2, see Table 1). To bring the carbonyl group in an appropriate position, a rotation around the C1–N2 bond has taken place (torsion angle N1–C1–N2–N6 = 35.9°), by which N6 was displaced above the central (CN_3_)^+^ plane; in contrast, the positions of all outer nitrogen atoms of the triaminoguanidinium core in salt **7a** deviate only little from the (CN_3_)^+^ plane.

**Table 1:** Hydrogen bonds in the solid-state structures of **7a** and **8** (Å and °).^a^

D–H···A	*d*(D–H)	*D*(H···A)	*d*(D···A)	<(DHA)

Salt **7a**				
N3–H3···Cl	0.84(3)	2.28(3)	3.102(2)	166(3)
N6–H6···Cl	0.90(3)	2.41(3)	3.275(2)	160(3)
N9–H9···Cl	0.86(3)	2.37(3)	3.205(2)	163(3)
N1–H1···N10	0.85(3)	2.04(3)	2.850(3)	159(3)
N4–H4···N11	0.83(3)	2.12(3)	2.915(4)	159(3)
N7-H7···O1^i^	0.90(3)	1.84(3)	2.709(3)	162(3)
Guanidine **8**				
N2–H2···N8	0.88(2)	2.25(2)	2.694(2)	111.4(13)
N3–H3···O1^ii^	0.88(2)	1.93(2)	2.771(2)	157.8(14)
N5–H5···O2	0.83(2)	2.31(2)	3.114(2)	163.8(17)
N7–H7···N1^iii^	0.89(2)	2.21(2)	3.085(2)	168.2(16)
N9–H9···O2	0.91(2)	2.05(2)	2.922(2)	160.6(17)

^a^See Figure 1 and Figure 2 for atom numbering. D = donor atom, A = acceptor atom. Symmetry codes: i = 2−*x*, 1−*y*, −*z*; ii = 2−*x*, 2−*y*, 1−*z*; iii = 1−-*x*, 2−*y*, 1−*z*.

**Figure 3:**
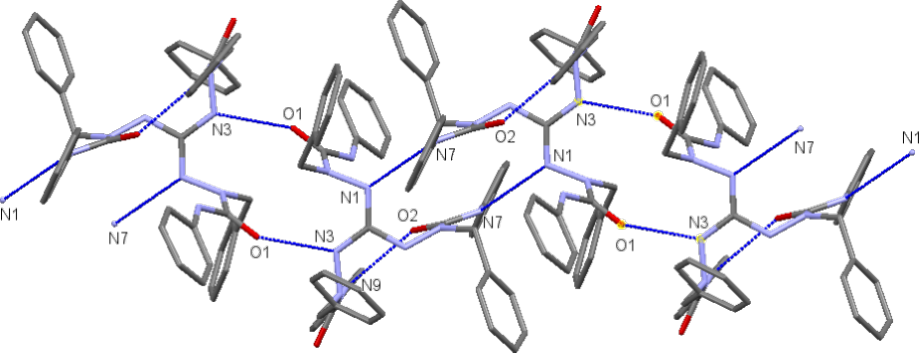
Hydrogen-bonded one-dimensional network of guanidine **8** in the solid state. The intramolecular N9···O2 hydrogen bond is also shown.

In the solid-state structures of both **7a** and **8**, all N–H hydrogen atoms are involved in hydrogen bonds. In salt **7a**, besides the three intramolecular N–H···Cl bonds already mentioned, two of the guanidinium N–H bonds maintain hydrogen bonds to two out of the three acetonitrile molecules in the asymmetric unit of the cell, and the third N–H bond forms an intermolecular N7–H···O1(=C) bond, which is part of a 14-membered ring in a centrosymmetric dimer (Figure 1, right). For guanidine derivative **8**, we have already mentioned the three intramolecular hydrogen bonds of N2–H, N5–H, and N9–H. In addition, each guanidine molecule forms two centrosymmetric dimers via N3–H···O1(=C) and N7–H···N1(=C) hydrogen bonds incorporated in 14-membered rings. In this manner, a one-dimensional chain is formed, which extends in the direction of the crystallographic *a* axis (Figure 3).

The NMR spectra (^1^H and ^13^C) of salts **7a**–**c** are quite simple. In agreement with the obvious threefold constitutional symmetry, all three branches of the cation give rise to the same set of signals (Table 2). Notably, the protons of the benzylic CH_2_ group are found to be diastereotopic, that is, the cation permanently exists in a chiral conformation; severe steric hindrance obviously excludes a conformation with all carbon and nitrogen atoms in a common plane. In contrast to the spectra of **7a**–**c**, those of **7d**–**f** are characterized by strong line broadening of the majority of signals for the nuclei of the cation over a wide temperature range (spectra were recorded between 295 and 373 K). This indicates conformational changes in the cation on the NMR time scale, which likely reside primarily in restricted rotation around the (NH)CH_2_–CH(CH_3_,Ph) carbon bond of the three branches.

**Table 2:** Selected ^1^H and ^13^C NMR data of salts **7** (in DMSO-*d*_6_, 295–300 K, δ [ppm] vs TMS).

	^1^H NMR			^13^C NMR
Salt	NCH_2_	NHCO	C^+^NH	NCH_2_

**7a**	3.81, 4.64	10.40	11.55	52.17
**7b**	3.75, 4.63	10.39	11.61	52.19
**7c**	3.82, 4.64	10.27	11.47	52.26
**7d**	3.61, 3.65^a,b^	8.56^c^	10.33^d^	58.0^c^
**7e**	3.56, 3.93^a,b^	8.74^c^	10.16^d^	57.7^c^
**7f**	3.57, 3.90^a,b^	8.39^c^	9.80^d^	58.0^c,e^

^a^At 373 K. ^b^Two broad, partially overlapping signals. ^c^Strongly broadened signal. ^d^Signal in coalescence. ^e^In CD_3_CN at 323 K.

The transition from salt **7a** to the neutral guanidine **8** is accompanied by a loss of the *C*_3_-symmetry of guanidinium salt **7a**. This becomes obvious when inspecting the ^1^H and ^13^C NMR spectra. However, these spectra were dynamic in various solvents and over a wide temperature range, and therefore, a complete assignment of most of these spectra was not possible. Due to the likely occurrence of several dynamic processes in solution (conformational changes with hindered rotation around single bonds, prototropic tautomerism in the guanidine moiety [1], dynamics of intra- and intermolecular NH hydrogen bonds), broad and partially overlapping signals and coalescence phenomena were observed. We were lucky, however, to obtain two spectra which clearly showed that the three branches of guandine **8** are magnetically non-equivalent: a) a ^1^H NMR spectrum taken in acetone-*d*_6_ at 290 K (see Supporting Information File 1) showed the presence of three AB spin systems for the NCH_2_ methylene protons and four signals for the five NH protons (3 × 1 H, 1 × 2 H); b) a ^13^C NMR spectrum recorded in DMSO-*d*_6_ at 373 K showed three NCH_2_ signals, while the room temperature spectrum of the same sample (as well as a spectrum taken in acetone-*d*_6_ at 295 K) showed only two of them.

### Reaction of salt **3** with isothiocyanates

**Scheme 5:**
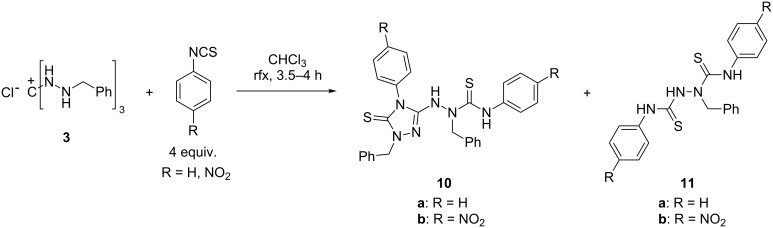
Reaction of 1,2,3-trisbenzylaminoguanidinium chloride (**3**) with aryl isothiocyanates.

**Figure 4:**
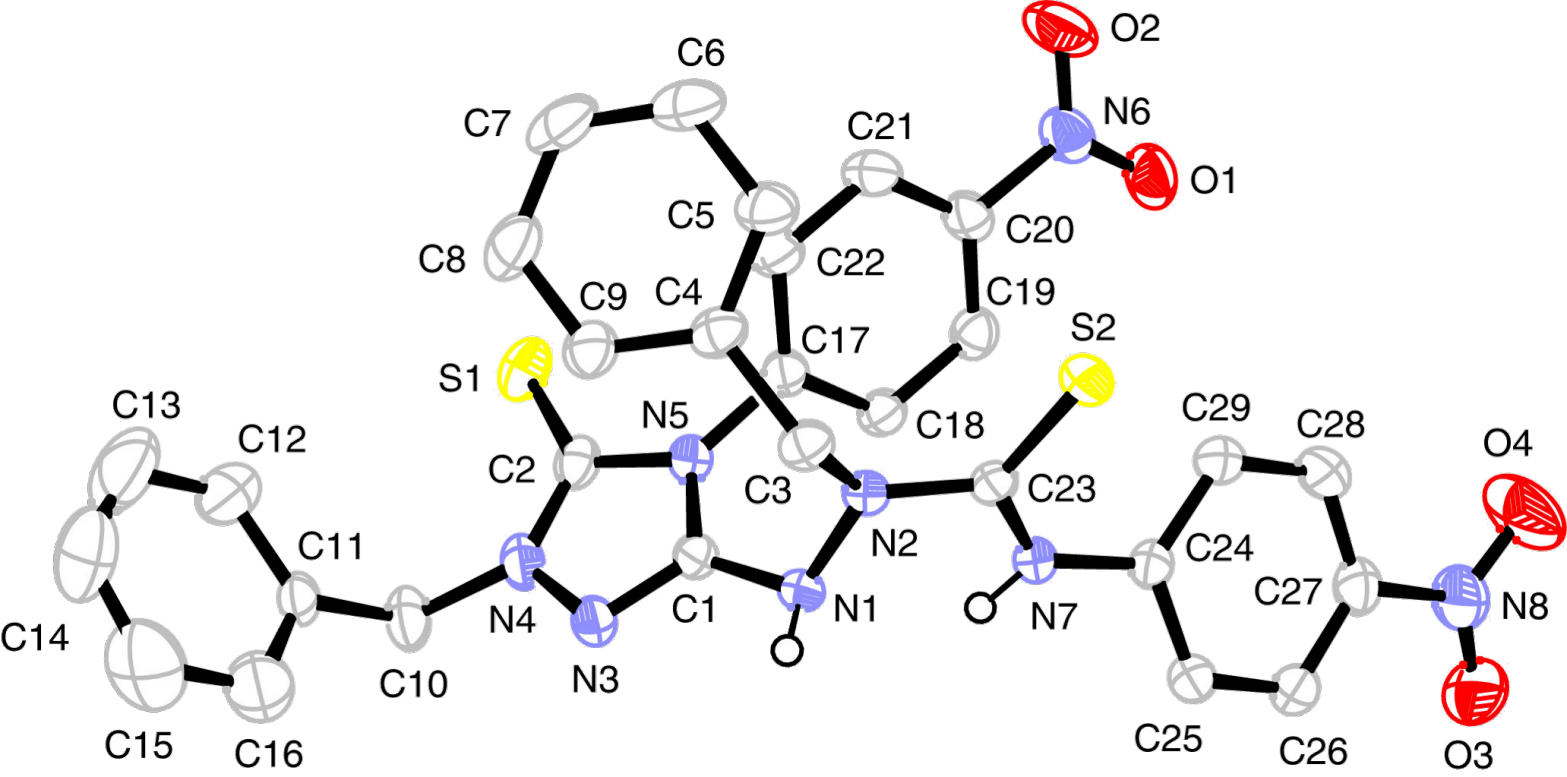
Solid-state structure of **10b**. Thermal displacement ellipsoids are drawn at the 30% probability level. Selected bond lengths (Å): C2–S1 1.664(2), C2–N4 1.346(3), C2–N5 1.382(3), N3–N4 1.381(3), C1–N1 1.388(3), C1–N3 1.298(2), C1–N5 1.374(2), C23–S2 1.672(2), C23–N2 1.377(2), C23–N7 1.353(3). Hydrogen bonds: a) N1···N7: N7–H 0.84 Å, N7···N1 2.584 Å, H···N1 2.12 Å, 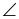 N7–H···N1 114.9°; b) N1···O1 (*x*, *y*+1, *z*): N1–H 0.85 Å, N1···O1 3.178 Å, H...O1 2.41 Å, 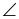 N1–H···O1 150.8°.

We thought that when salt **3** was combined with at least three molar equivalents of an isothiocyanate instead of an isocyanate, likewise a threefold sulfonylcarbamoylation, with formation of a tris(thiourea) derivative, could result. With phenyl isothiocyanate and its 4-nitro derivative, however, 3-hydrazinyl-1*H*-1,2,4-triazole-5(4*H*)-thiones **10a,b** were obtained as major products in 57 and 68% yield, respectively (Scheme 5). In the case of (4-nitrophenyl) isothiocyanate, the bis(arylthiourea) **11b** was isolated as a byproduct in 26% yield. The corresponding bis(arylthiourea) **11a** could not be isolated, although its formation had been suggested by NMR spectra of the reaction mixture. The molecular structure of **10b** was established by an X-ray diffraction analysis and is shown in Figure 4. The unsymmetrical constitution of bis(arylthiourea) **11b** became evident from the ^13^C NMR spectrum which showed the expected number of signals; further support for the proposed structure was provided by HSQC and HMBC H,C correlation spectra.

**Scheme 6:**
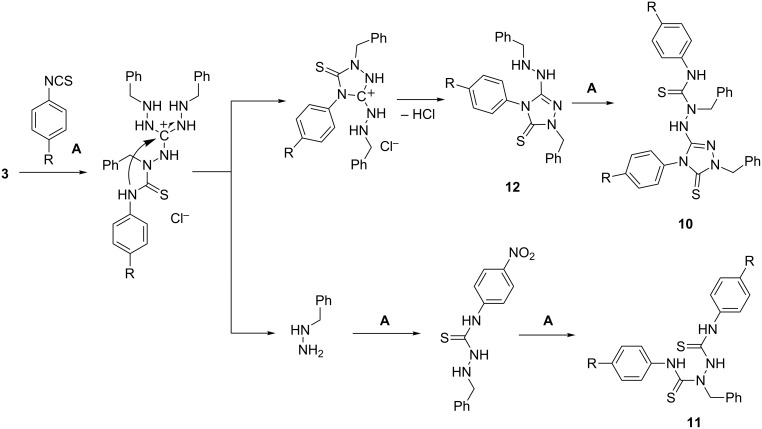
Proposed mechanism of the formation of **10** and **11**.

A mechanistic proposal for the formation of compounds **10** and **11** is shown in Scheme 6. We assume that the reaction starts with the thiocarbamoylation of one of the benzyl-substituted amino functions of **3**. Cyclization of the formed intermediate with participation of the thiourea-NH nitrogen gives rise to 1,2,4-triazole-5(4*H*)-thione **12** from which the major reaction product **10** is formed in a second thiocarbamoylation step. Benzylhydrazine, which is liberated in the course of the cyclization step, is transformed into byproduct **11** by thiocarbamoylation of both nitrogen atoms with the aryl isothiocyanate. The formation of triazole **12** is related to the reaction of TAG-NO_3_ with phenyl- and allyl isothiocyanate [13].

## Conclusion

In summary, we have developed a three-step synthetic sequence by which 1,2,3-triaminoguanidinium chloride (TAG-Cl) is converted into derivatives featuring double substitution at each of the three outer NH_2_ groups, namely 1,2,3-tris(1-alkyl-3-arylureido)guanidinium salts, which can easily be deprotonated to afford a neutral guanidine derivative. TAG-Cl is soluble only in water or some alcohol/water mixtures; this prevents the reaction with certain reactive electrophiles. Our method includes the conversion of TAG-Cl into 1,2,3-tris(iminyl)guanidinium salts, which are reduced to form 1,2,3-tris(alkylamino)guanidinium salts, and finally *N*-carbamoylation with arylisocyanates. With (*p*-toluenesulfonyl) isocyanate only a *N*-mono-sulfonylcarbamoylation could be achieved. In contrast, 3-hydrazinyl-1*H*-1,2,4-triazole-5(4*H*)-thiones were obtained from 1,2,3-tris(benzylamino)guanidinium chloride and aryl isothiocyanates. Here, a cyclization reaction occurred after thiocarbamoylation of the first NH_2_ group. The diverse reaction routes reported in this paper nicely demonstrate the versatility of TAG-derived 1,2,3-tris(alkylamino)guanidinium ions respect to synthetic transformations using heterocumulenes as reagents. Due to their particular molecular shape and the presence of hydrogen-bond donor and acceptor groups, the obtained 1,2,3-tris(ureido)guanidinium salts **7** and the derived neutral guanidine **8** may become of interest as host components in host–guest complexes, for biological activity studies, and in crystal engineering.

## Supporting Information

File 1 Experimental procedures, characterization data for synthesized compounds, and data for the X-ray crystal structure determinations.
