# Supplementary material for: Derivatives of the triaminoguanidinium ion, 3. Multiple N-functionalization of the triaminoguanidinium ion with isocyanates and isothiocyanates
Source: Beilstein J Org Chem. 2014 Sep 24;10:2255–62. doi: 10.3762/bjoc.10.234 (PMC4187099; doi:10.3762/bjoc.10.234)
Supplement: File 1 — Experimental procedures, characterization data for synthesized compounds, and data for the X-ray crystal structure determinations. [file Beilstein_J_Org_Chem-10-2255-s001.pdf]

Supporting Information

for

Derivatives of the triaminoguanidinium ion, 3. Multiple *N*-functionalization of the triaminoguanidinium ion with isocyanates and isothiocyanates

Jan Szabo[1], Kerstin Karger[1], Nicolas Bucher[1,2] and Gerhard Maas*[1]

Address: [1]Institute of Organic Chemistry I, University of Ulm, Albert-Einstein-Allee 11, D-89081 Ulm, Germany, Fax: (+49) 731-50-22803 and [2](new address) TUM CREATE, Singapore 138602, Singapore

Email: Gerhard Maas* - gerhard.maas@uni-ulm.de

* Corresponding author

Experimental procedures, characterization data for synthesized compounds, and data for the X-ray crystal structure determinations

Table of contents

Experimental procedures and characterization of compounds S2

X-ray crystal structure determinations S16

References S24

Experimental details and characterization of compounds

General information

^{1}H and ^{13}C NMR spectra were recorded on Bruker Avance 400 (^{1}H: 400.13 MHz; ^{13}C: 100.62 MHz) and Bruker Avance 500 spectrometers (^{1}H: 500.14 MHz, ^{13}C: 125.76 MHz); δ values are reported in ppm and coupling constants are expressed in Hertz (Hz) (m_c = centered multiplet). The signal of the solvent was used as internal standard: ^{1}H spectra: $\delta(CHCl_3)$ = 7.26, $\delta((CH_3)_2SO)$ = 2.50, $\delta(CH_3CN)$ = 1.94 ppm; ^{13}C spectra: $\delta(CDCl_3)$ = 77.0, $\delta((CD_3)_2SO)$ = 39.43, $\delta(CD_3CN)$ = 1.24 ppm. NMR spectra were measured at 293±2 K if not stated otherwise. When necessary, ^{13}C signal assignments were derived from C,H COSY, HSQC and HMBC spectra. IR spectra: Bruker Vector 22; wave numbers [cm^{-1}] and intensities (vs = very strong, s = strong, m = medium, w = weak, br = broad) are given. Elemental analyses: elementar vario MICRO cube. Mass spectra: Bruker Daltonics REFLEX III (MALDI-TOF spectra, detection N_2 laser with 337 nm, matrix *trans*-2-(3-(4-*tert*-butylphenyl)-2-methyl-2-propenylidene)malononitrile), Bruker solariX (MALDI-TOF and ESI HRMS-spectra). Column chromatography was performed under hydrostatic pressure (silica gel Si 60, Macherey-Nagel, 0.063–0.2 mm). Melting points were determined with a Büchi Melting Point B-540 apparatus. The reactions were carried out under air atmosphere unless otherwise noted.

Materials. Phenyl isocyanate was purchased from ACROS, phenyl isothiocyanate from Fluka and *p*-toluenesulfonyl isocyanate from ABCR. Triaminoguanidium chloride (**1**) [1], 1,2,3-tris(benzylamino)guanidinium chloride (**3**) [2], (4-bromophenyl) isocyanate [3] and (4-methylphenyl) isothiocyanate [3] were prepared by published procedures.

Synthetic procedures

1,2,3-Tris(2-phenylpropyl-1-iminyl)guanidinium chloride (**4**): Triaminoguanidinium chloride (**1**) (10.0 g, 71.4 mmol) was dissolved in 100 mL of water and hydratropic aldehyde (29.4 mL, 221.3 mmol) was added. While the reaction mixture was kept in an ultrasonic bath for 2 h, a red viscous oil deposited, which was extracted several times with *tert*-butyl methyl ether and diethyl ether until it became colorless. After drying of the oil at 22 ^{o}C/0.05 mbar, a colorless solid was obtained (25.1 g, 51.3 mmol, 72 %), m.p. 134.8-135.2 °C. – IR (KBr): ν = 3650–2250 (continous absorption with medium-strong maxima at 3617, 3314, 3027, 2971, 2930, 2873), 1952 (w), 1878 (w), 1810 (w), 1618 (vs, br, C=N), 1492 (s), 1451 (s), 1311 (s), 1099 (s), 1051 (s), 1019 (s), 762 (s), 700 (s) cm^{-1}. – ^{1}H NMR ($(CD_3)_2SO$, 400.13 MHz): δ = 1.45 (d, ^{3}J = 6.2 Hz, 9 H, $CHCH_3$), 3.80 (m_c, 3 H, $CHCH_3$), 7.1–7.3 (m, 15 H, H_{Ph}), 8.11 (d, ^{3}J = 5.2 Hz, 3 H, CH=N), 11.68 (s, 3 H, $C^{+}NH$) ppm. – ^{13}C NMR ($(CD_3)_2SO$, 100.62 MHz): δ = 18.49 (CH_3), 42.13 (CH), 126.97 (*p*-CH_{Ph}), 127.46 (CH_{Ph}), 128.80 (CH_{Ph}), 141.73 (*ipso*-C_{Ph}), 149.16 (C=N), 157.93 ($C^{+}(NH)_3$) ppm. – MS (MALDI-TOF): *m/z* = 453 $[M – Cl]^{+}$. – Anal. calcd. for $C_{28}H_{33}ClN_6$ (489.06): C, 68.77; H, 6.80; N, 17.18; found: C, 68.78; H, 6.93; N, 17.19.

1,2,3-Tris(2-phenylpropyl-1-amino)guanidinium tosylate (**5-OTs**): 1,2,3-Tris(2-phenylpropyl-1-iminyl)guanidinium chloride (**4**) (3.92 g, 8.0 mmol) and dimethylaminoborane (2.27 g, 38.5 mmol) were placed in a reaction flask and dichloromethane (50 mL) was added. A large excess of *p*-toluenesulfonic acid (33.15 g, 192.5 mmol) dissolved in CH_2Cl_2 and MeOH (3:1, 10 mL) was slowly added to the suspension (gas evolution) while stirring at room temperature. After 1.5 h a saturated solution of Na_2CO_3 (30 mL) was added, and the mixture was stirred for another hour.

The organic phase was separated, and the aqueous phase was extracted with CH_2Cl_2. The combined organic layers were dried over Na_2SO_4 and the solvent was evaporated at 0.05 mbar/20 ^oC to leave a yellow, highly viscous oil, which was sufficiently pure for further transformations (4.26 g, 84 %). – IR (KBr): ν = 3425 (broad, m), 3273 (broad, s), 3060 (m), 3027 (m), 2962 (m), 2927 (m), 2871 (m), 1948 (w), 1876 (w), 1806 (w), 1653 (vs), 1602 (m), 1494 (m), 1453 (m), 1381 (w), 1193 (s), 1122 (m), 1033 (m), 1011 (m), 949 (w), 912 (w), 864 (w), 816 (w), 762 (m), 701 (vs), 682 (m), 566 (m) cm^{-1}. – ^1H NMR ($(CD_3)_2SO$, 500.16 MHz, 300 K): δ = 1.22-1.24 (m, 9 H, $CHCH_3$), 2.28 (s, 3 H, aryl-CH_3), 2.78–2.82 (m, 6 H, $CHCH_2$), 2.86–2.97 (m, 3 H, $CHCH_2$), 4.95–5.00 (m, 2 H, NH), 5.02–5.04 (m, 1 H, NH), 7.12/7.47 (AA'BB' spin system, ^3J = 7.9 Hz, 4 H, H_{Ar}), 7.16–7.20 (m, 8 H, H_{Ph}), 7.26–7.30 (m, 7 H, H_{Ph}), 8.30 (broadend s, 3 H, C^+NH) ppm. – ^1H NMR ($(CD_3)_2SO$, 500.16 MHz, 323 K): δ = 1.23 (d, ^3J = 6.5 Hz, 9 H, $CHCH_3$), 2.29 (s, 3 H, aryl-CH_3), 2.80–2.84 (m, 6 H, $CHCH_2$), 2.87–2.93 (m, 3 H, $CHCH_2$), 4.86 (s, 3 H, NH), 7.11/7.49 (AA'BB' spin system, ^3J = 7.9 Hz, 4 H, H_{Ar}), 7.17–7.21 (m, 8 H, H_{Ph}), 7.25–7.30 (m, 7 H, H_{Ph}), 8.04 (s, 3 H, C^+NH) ppm. – ^{13}C NMR ($(CD_3)_2SO$, 300 K): δ = 20.11 (CH_3), 20.77 (CH_3), 37.46 ($CHCH_3$), 57.69 (NCH_2CH), 125.48 (C_{Ar}), 126.20 (p-C_{Ph}), 126.93 (CH_{Ph}), 128.01 (C_{Ar}), 128.40 (CH_{Ph}), 137.53 (C_{Ar}), 145.20 (C_{Ph}), 145.79 (C_{Ar}), 157.16 ($C^+(NH)_3$) ppm. - MS (MALDI-TOF): *m/z* = 459 $[M – OTs]^+$. – Anal. calcd. for $C_{35}H_{46}O_3S$ (630.85): C, 66.64; H, 7.35; N, 13.32; S 5.08; found: C, 66.83; H, 7.29; N, 13.51; S, 4.93.

1,2,3-Tris(2-phenylpropyl-1-amino)guanidinium chloride (**5-Cl**): 1,2,3-Tris(2-phenylpropyl-1-iminyl)guanidinium chloride (**4**) (0.502 g, 1.00 mmol) and dimethylaminoborane (0.282 g, 4.80 mmol) were placed in a reaction flask, and dichloromethane (20 mL) and hydrochloric acid (37 %, 7 mL) were added. The

suspension was stirred at room temperature, until the gas evolution had ceased (90 min). Work-up as described above for **5-OTs** afforded a highly viscous yellow oil, which could not be purified further (0.466 g, 90 %). – IR (KBr): ν = 3670–2640 (continuous absorption with distinct maxima at 3424 (s), 3060 (m), 3027 (m), 2967 (m), 2928 (m), 2872 (m)), 1949 (w), 1876 (w), 1807 (w), 1633 (vs), 1599 (vs), 1542 (m), 1493 (m), 1451 (m), 1380 (m), 1126 (w), 1018 (w), 912 (w), 762 (m), 700 (s) cm^{-1}. – ^1H NMR ($(CD_3)_2SO$, 500.16 MHz, 300 K): δ = 1.22–1.24 (m, 9 H, $CHCH_3$), 2.77–2.82 (m, 6 H, $CHCH_2$), 2.86–2.90 (m, 3 H, $CHCH_2$), 5.01 (s, 2 H, NH), 5.04 (s, 1 H, NH), 7.16–7.29 (m, 15 H, H_{Ph}), 8.45 (s, 3 H, C^+NH) ppm. – ^1H NMR ($(CD_3)_2SO$, 500.16 MHz, 323 K): δ = 1.23 (d, ^3J = 6.5 Hz, 9 H, $CHCH_3$), 2.79–2.85 (m, 6 H, $CHCH_2N$), 2.89 (narrow m, 3 H, $CHCH_2$), 4.94 (s, 3 H, $NHCH_2$), 7.17–7.30 (m, 15 H, H_{Ph}), 8.39 (s, 3 H, C^+NH) ppm. – ^{13}C NMR ($(CD_3)_2SO$, 100.62 MHz, 295 K): δ = 20.13 ($CHCH_3$), 37.47 ($CHCH_3$), 57.76 (NCH_2), 126.19 (p-C_{Ph}), 126.98 (CH_{Ph}), 128.41 (CH_{Ph}), 145.29 (C_{Ph}), 157.18 ($C^+(NH)_3$) ppm. – MS (MALDI-TOF): *m/z* = 459 $[M - Cl]^+$. – Anal. calcd. for $C_{28}H_{39}ClN_6$ (496.11): C, 67.79; H, 8.13; N, 16.94; found: C, 67.10; H, 7.50; N, 16.07. The salt could not be obtained in analytically pure form.

*General procedure for the carbamoylation of salt **3*** (products **7a**–**c**): Salt **3** (8.20 g, 20.0 mmol) was suspended in anhydrous CH_2Cl_2 (400 mL) under an argon atmosphere. A solution of an isocyanate **6a**–**c** (66 mmol) in anhydrous CH_2Cl_2 (120 mL) was added slowly. The reaction mixture was stirred for the time and at the temperature given for the individual compounds. The formed voluminous precipitate was collected by filtration, washed with CH_2Cl_2 and dried (120 °C/0.05 mbar). Salts **7a**–**c** were found to be well soluble in methanol, acetone, and dimethyl sulfoxide.

1,2,3-Tris(1-benzyl-3-phenylureido)guanidinium chloride (**7a**): From **3** and **6a**. Stirring for 24 h at room temperature afforded a brittle white solid (13.98 g, 91 %), m. p. 183.3–186.2 °C. – IR (KBr): ν = ~3550–2000 (little structured continuous absorption with a strong absorption band at 3033), 1674 (s), 1600 (s), 1546 (s), 1499 (s), 1445 (s), 1321 (m), 1247 (m), 1076 (w), 1028 (w), 933 (w), 899 (w), 854 (w), 750 (s), 692 (s), 650 (m), 592 (w), 500 (w), 437 (w) cm^{-1}. – ^1H NMR ($(CD_3)_2SO$, 400.13 MHz): δ = 3.81 (d, ^2J = 15.2 Hz, 1 H, $PhCH_AH_B$), 4.64 (d, ^2J = 15.2 Hz, 1 H, $PhCH_AH_B$), 7.02 (apparent s, 1 H, H_{Ph}), 7.28 (apparent s, 7 H, H_{Ph}), 7.90 (apparent s, 2 H, H_{Ph}), 10.40 (s, 1 H, CO-NH), 11.55 (s, 1 H, C^+NH) ppm. – ^{13}C NMR ($(CD_3)_2SO$, 100.62 MHz): δ = 52.17 (NCH_2), 120.36 (CH_{Ph}), 122.77 (CH_{Ph}), 127.50 (CH_{Ph}), 128.25 (CH_{Ph}), 128.31 (CH_{Ph}), 128.33 (CH_{Ph}), 135.96 (C_{Ph}), 139.58 (C_{Ph}), 155.02 (C=O), 157.47 ($C^+(NH)_3$) ppm. – Anal. calcd. for $C_{43}H_{42}ClN_9O_3$ (767.31): C, 67.22; H, 5.51; N, 16.41; found: C, 67.10; H, 5.49; N, 16.32.

1,2,3-Tris(1-benzyl-3-(4-bromophenyl)ureido)guanidinium chloride (**7b**): From **3** and **6b**. Stirring for 30 min at 55 °C afforded a brittle white solid (17.48 g, 87 %), m. p. 201 °C (dec.). – IR (KBr): ν = 3296 (br. m), 3031 (m), 1676 (s), 1634 (s), 1593 (s), 1538 (s), 1455 (m), 1399 (s), 1313 (m), 1241 (m), 1074 (m), 1011 (m), 924 (w), 821 (m), 755 (m), 701 (m) cm^{-1}. – ^1H NMR ($(CD_3)_2SO$, 400.13 MHz): δ = 3.75 (d, ^2J = 15.6 Hz, 1 H, $PhCH_AH_B$), 4.63 (d, ^2J = 15.6 Hz, 1 H, $PhCH_AH_B$), 7.22–7.27 (m, 5 H, H_{Ar}), 7.48 (d, J = 8.5 Hz, 2 H, H_{Ar}), 7.85 (d, J = 7.8 Hz, 2 H, H_{Ph}), 10.39 (s, 1 H, CO-NH), 11.61 (s, 1 H, C^+NH) ppm. – ^{13}C NMR ($(CD_3)_2SO$, 100.62 MHz): δ = 52.19 (NCH_2), 114.64 (C_{Ar}-Br); 122.14, 127.64, 128.25, 128.43, 131.26 (all CH_{Ph} and CH_{Ar}); 135.76, 139.96 (*ipso*-C_{Ph} and -C_{Ar}); 154.81 (C=O), 157.45 ($C^+(NH)_3$) ppm. – Anal. calcd. for $C_{43}H_{39}Br_3ClN_9O_3$ (1004.99): C, 51.39; H, 3.91; N, 12.54; calcd. for $C_{43}H_{39}Br_3ClN_9O_3 \times 1\ H_2O$: C, 50.46; H, 4.04; N, 12.31; found: C, 50.47; H, 4.08; N, 12.29.

1,2,3-Tris(1-benzyl-3-(4-methylphenyl)ureido)guanidinium chloride (**7c**): From **3** and **6c**. Stirring for 4.5 h at 69 °C afforded a brittle white solid (14.26 g, 91 %), m. p. 184.7–185.9 °C. – IR (KBr): ν = 3397 (m), 3284 (br. m), 3139 (br. m), 3033 (m), 2921 (m), 1666 (s), 1603 (s), 1518 (s), 1455 (m), 1409 (m), 1319 (m), 1245 (m), 1110 (w), 1073 (w), 1029 (w), 921 (w), 813 (m), 757 (m), 700 (m) cm^{-1}. – ^1H NMR ($(CD_3)_2SO$, 500.16 MHz): δ = 2.25 (s, 3 H, CH_3), 3.82 (d, ^2J = 15.5 Hz, 1 H, $PhCH_AH_B$), 4.58 (d, ^2J = 15.5 Hz, 1 H, $PhCH_AH_B$), 7.08/7.75 (AA'BB' spin system, ^3J = 8.1 Hz, 4 H, C_6H_4-4-CH_3), 7.22–7.27 (m, 5 H, H_{Ph}), 10.27 (s, 1 H, CO-NH), 11.47 (s, 1 H, C^+NH) ppm. – ^{13}C NMR ($(CD_3)_2SO$, 125.76 MHz): δ = 20.48 (aryl-CH_3), 52.26 (NCH_2); 120.40, 127.48, 128.28, 128.32, 128.77 (all CH_{Ph} and CH_{Ar}); 131.59, 136.03, 137.00 (C_{Ph} and C_{Ar}); 155.08 (C=O), 157.53 ($C^+(NH)_3$) ppm. – Anal. calcd. for $C_{46}H_{48}ClN_9O_3$ (810.38): C, 68.18; H, 5.97; N, 15.56; found: C, 68.15; H, 5.97; N, 15.72.

General procedure for the carbamoylation of salt ***5-OTs*** (products **7d–f**): Salt **5-OTs** (0.50 g, 0.8 mmol) was suspended in anhydrous CH_2Cl_2 (20 mL) under an argon atmosphere, and a solution of an isocyanate **6a–c** (2.6 mmol) in anhydrous CH_2Cl_2 (10 mL) was added slowly with stirring. A clear solution developed gradually. After cooling, a white solid was precipitated by addition of pentane, filtered off, washed with pentane, and dried at 50 °C/0.05 mbar.

1,2,3-Tris(1-(2-phenylpropyl)-3-phenylureido)guanidinium tosylate (**7d**): The reaction mixture of **5-OTs** and **6a** in CH_2Cl_2 was stirred at room temperature for 14 h. After work-up a white solid was obtained (0.71 g, 90 % yield), m. p. 173.8–174.3 °C. – IR (KBr): ν = ~ 3570–2500 (continuous absorption with distinct maxima at 3289, 3135, 3058, 3029, 2966, 2931), 1944 (w), 1872 (w), 1777 (m), 1673 (s), 1597 (s), 1549 (s), 1499 (s), 1443 (s), 1383 (m), 1314 (s), 1231 (s), 1204 (s), 1120 (m), 1031 (m), 1011

(m), 896 (m), 813 (m), 754 (s), 695 (s) cm^{-1}. – ^1H NMR (CD_3CN, 400.13 MHz): δ = 1.12–1.37 (m, 9 H, CHCH_3), 2.27 (s, 3 H, aryl-CH_3), 2.85–4.35 (m, 9 H, CHCH_2), ~ 6.7–7.6 (m, 30 H, H_{Ph}), 7.04/7.60 (AA'BB' spin system, ^3J = 8.1 Hz, 4 H, C_6H_4), ~ 8.1–10.0 (two very broad signals, 3 H, NH) ppm. ^1H NMR ($(CD_3)_2SO$, 500.16 MHz, 373 K): δ = 1.35 (broadened s, 9 H, CHCH_3), 2.30 (s, 3 H, aryl-CH_3), 3.16 (broadened s, 3 H, C$H$$CH_2$), 3.61 and 3.95 (two broadened, unstructured signals, 6 H, CHCH_2), 7.0–7.5 (m, 30 H, H_{Ph}), 7.09/7.56 (AA'BB' spin system, ^3J = 7.9 Hz, 4 H, C_6H_4 of tosylate), 8.56 (broadened s, 3 H, NH), 10.33 (very broad coalescing signal, 3 H, NH) ppm. – ^{13}C NMR (CD_3CN, 125.76 MHz, 320 K): δ = 20.64 (coalescing signal, CH$C$$H_3$), 21.40 (aryl-$CH_3$), 38.88 ($CHCH_2$), 58.0 (coalescing, CH$C$$H_2$N), 120.10 ($C_{Ph}$), 121.72 (broad, C_{Ph}), 124.79 (broad, C_{Ph}), 126.86 (tosylate-CH), 127.88 (broadened, C_{Ph}), 128.44 (tosylate-CH), 129.69 (C_{Ph}), 129.72 (C_{Ph}), 139.61 (broad, C_{Ph}), 140.78 (tosylate-C), 144.94 (tosylate C), 145.39 (coalescing, C_{Ph}), 156.61 (broad, coalescing signal, $C^+(NH)_3$ or C=O, the second signal was not detected) ppm. – MS (MALDI-TOF): m/z = 816.9 $[M - OTs]^+$. – Anal. calcd. for $C_{56}H_{61}N_9O_6S$ (988.22): C, 68.06; H, 6.22; N, 12.76; S, 3.24; found: C, 67.88; H, 6.03; N, 12.71; S, 2.93.

1,2,3-Tris(1-(2-phenylpropyl)-3-(4-bromophenyl)ureido)guanidinium tosylate (**7e**): The reaction mixture **5-OTs** and **6b** in CH_2Cl_2 was stirred at room temperature for 20 h. After work-up a white solid was obtained (0.76 g, 78 % yield), m. p. 148.1–148.9 °C. – IR (KBr): ν = ~ 3700–2250 (continuous absorption with medium-strong maxima at 3291, 3029, 2968, 2930), 1676 (s), 1595 (s), 1535 (vs), 1492 (vs), 1453 (m), 1400 (s), 1310 (m), 1286 (m), 1237 (s), 1180 (s), 1122 (m), 1074 (m), 1036 (m), 1011 (m), 820 (m), 762 (m), 702 (m), 683 (m) cm^{-1}. – ^1H NMR ($(CD_3)_2SO$, 400.13 MHz): δ = 1.23–1.45 (m, 9 H, CHCH_3), 2.29 (s, 3 H, aryl-CH_3), 2.99–3.14 (m, 3 H, C$H$$CH_2$),

3.52–4.26 (m, 6 H, NCH_2), 6.95–7.60 (m, 31 H, H_{Ph} and H_{Ar}), 8.68–9.27 (m, 3 H, NH), 10.39–11.54 (m, 3 H, NH) ppm. – ^{1}H NMR ($(CD_3)_2SO$, 500.16 MHz, 373 K): δ = 1.31 (s, 9 H, $CHCH_3$), 2.30 (s, 3 H, aryl-CH_3), 3.13 (broad "s", 3 H, $CHCH_2$), 3.56 and 3.93 (two broad, unstructured and overlapping signals, 6 H, $CHCH_2$), ~ 7.10–7.60 (m, 27 H, $H_{Ph,}$ H_{Ar}), 7.09/7.56 (AA'BB' spin system, ^3J = 7.1 Hz, 4 H, C_6H_4 of tosylate), 8.74 (broad coalescing signal, 3 H, NH), 10.16 (almost vanishing, coalescing signal for 3 NH) ppm. – ^{13}C NMR (CD_3CN, 125.76 MHz): δ = 20.35 (coalescing, $CHCH_3$), 21.33 (tosylate-CH_3), 38.54 (coalescing, $CHCH_2$), 57.7 (coalescing, $CHCH_2N$); 121.29, 123.06, 126.58 (tosylate-CH), 127.64, 128.20, 129.55 (tosylate-CH), 129.75, 132.36, 138.60, 140.88, 143.88 (tosylate-C), 144.92 (tosylate-C), 156.3 and 159.8 (broad signals, $C^+(NH)_3$ and C=O) ppm. – MS (MALDI-TOF): *m/z* = 1052.3 $[M – OTs]^+$. – Anal. calcd. for $C_{56}H_{58}Br_3N_9O_6S$ (1224.91): C, 54.91; H, 4.77; N, 10.29; found: C 54.77, H 4.83, N 10.32.

1,2,3-Tris(1-(2-phenylpropyl)-3-(4-methylphenyl)ureido)guanidinium tosylate (**7f**): The reaction mixture was stirred at room temperature for 46 h. After work-up a white solid was obtained (0.61 g, 74 % yield), m. p. 132.6–134.3 °C. – IR (KBr): ν = ~ 3700–2500 (continuous absorption with medium-strong maxima at 3301, 3030, 2967, 2924), 1672 (s), 1604 (s), 1518 (vs), 1453 (m), 1409 (m), 1318 (m), 1295 (w), 1240 (s), 1206 (m), 1176 (m), 1122 (m), 1035 (m), 1012 (m), 815 (m), 762 (m), 702 (m), 682 (m) cm^{-1}. – ^{1}H NMR (CD_3CN, 400.13 MHz): δ = 1.00–1.45 (m, 9 H, $CHCH_3$), 2.26 (s, 3 H, tosylate-CH_3, superimposing a broadened s, 9 H, tolyl-CH_3), 2.8–4.3 (two partially overlapping broad multiplets, 9 H, $CHCH_2$), 6.8–7.4 (m, 27 H, H_{Ph} and H_{tolyl}), 7.09/7.56 (AA'BB' spin system, ^3J = 8.1 Hz, 4 H, C_6H_4 of tosylate), 8.20–8.34 (m, 3 H, NH), 9.55 (s, 3 H, NH) ppm. ^{1}H NMR ($(CD_3)_2SO$, 500.16 MHz, 358 K): δ = 1.33 (slightly broadened s, 9 H, $CHCH_3$), 2.28 (s, 9 H, tolyl-CH_3), 2.30 (s, 3 H, tosylate-

CH_3), 3.14/3.57/3.90 (three broadened, partially overlapping signals, 3 H each, C*H*C*H*$_2$), 7.03 (AA' part of an AA'BB' spin system, *J* = 7.8 Hz, 6 H, 4-MeC_6H_4), 7.09/7.55 (AA'BB' spin system, ^3J = 8.1 Hz, 4 H, C_6H_4 of tosylate), 7.14–7.43 (m, 21 H, 15 H_{Ph} and 6 H_{BrC6H4}), 8.39 (broadened s, 3 H, NH), 9.80 (very broad, almost vanishing signal in coalescence, for 3 NH) ppm. – ^{13}C NMR (CD_3CN, 125.76 MHz, 320 K): δ = 20.46 (CH_3), 20.98 (CH_3), 21.43 (CH_3), 38.98 (*C*HCH_2), 58.0 (coalescing, CH*C*H_2N); 121.80, 126.90 (tosylate-CH), 127.90, 128.48, 129.69 (tosylate-CH), 129.88, 130.19, 134.51, 137.07, 140.69 (tosylate-C), 145.18, 145.56 ppm (tosylate-C); 156.78 (coalescing, $C^+(NH)_3$ or C=O, the second signal was not detected). – MS (MALDI-TOF): *m/z* = 858.6 $[M – OTs]^+$. – Anal. calcd. for $C_{59}H_{67}N_9O_6S$ (1030.30): C, 68.78; H, 6.55; N, 12.24; S, 3.11; found: C, 68.92; H, 6.54; N, 12.34; S, 2.86.

1,2,3-Tris(1-benzyl-3-phenylureido)guanidine (**8**): 1,2,3-Tris(1-benzyl-3-phenylureido)guanidinium chloride (**7a**) (2.00 g, 2.60 mmol) was dissolved in methanol/acetone (75/50 mL) at ambient temperature. On addition of an aqueous sodium hydroxide solution (1 M, 50 mL), a pale-yellow color developed. After ten minutes, the formed white precipitate was filtered off, washed with water, and freeze-dried (1.68 g, 89 % yield); m. p. 120.2–126.1 °C. – IR (KBr): ν = 3401 (w), 3307 (br, m), 3060 (w), 1675 (s), 1650 (vs), 1602 (s), 1516 (br, s), 1445 (s), 1327 (m), 750 (s), 693 (s) cm^{-1}. – ^{1}H NMR ($(CD_3)_2SO$, 400.13 MHz, 293 K): see Figure 1. ^{1}H NMR (D_6-acetone, 400.13 MHz, 295 K): δ = 4.05 (broad unstructured signal)/5.07 (d, *J* = 14.4 Hz) (2 H, NCH^ACH^B), 4.17/4.85 (AB spin system, ^2J = 14.0 Hz, 2 H, NCH^ACH^B), 4.59/4.67 (AB spin system with broadened lines, 2 H, NCH^ACH^B), 6.83–7.62 (several m, 30 H, H-phenyl), 8.07 (broadened, 1 H, NH), 8.26 (broadened, 2 H, NH), 8.51 (broadened, 1 H, NH), 8.60 (broadened, 1 H, NH) ppm. – ^{13}C NMR ($(CD_3)_2SO$, 100.62 MHz, 295 K): δ = 49.59 (broad, NCH_2), 53.25 (broad, NCH_2); 117.56, 121.50,

122.29, 123.07, 126.82, 126.92, 127.39, 127.81, 128.35, 128.59, 128.82, 129.24 (all CH_{Ph}); 135.99, 138.02, 139.29, 139.73 (all C_{Ph}), 155.41 (CO), 157.24 (C=N) ppm. ^{13}C NMR ($(CD_3)_2SO$, 100.62 MHz, 295 K): δ = 50.73, 51.07, 52.66 (3 NCH_2); 117.87 (broadened, 118.89 (broadened), 119.93 (broadened), 121.18, 121.86, 122.47, 126.16, 126.36, 126.88, 127.24, 127.51, 127.7 (broad, several signals), 128.16, 128.52 (all CH_{Ph}); 135.53, 138.6 (broad), 138.98 (broad) (all *ipso*-C_{Ph}); 155.2 (broad), 155.5 (broad), 156.73 (broadened) (C=O and $C^+(NH)_3$). – MS (MALDI-TOF): *m/z* = 732.3 $[M + H]^+$, 754.3 $[M + Na]^+$, 770.3 $[M + K]^+$, 613.3 [M – PhNCO]. – Anal. calcd. for $C_{43}H_{41}N_9O_3$ (731.33): C, 70.57; H, 5.65; N, 17.23; found: C, 70.57; H, 5.63; N, 17.07.

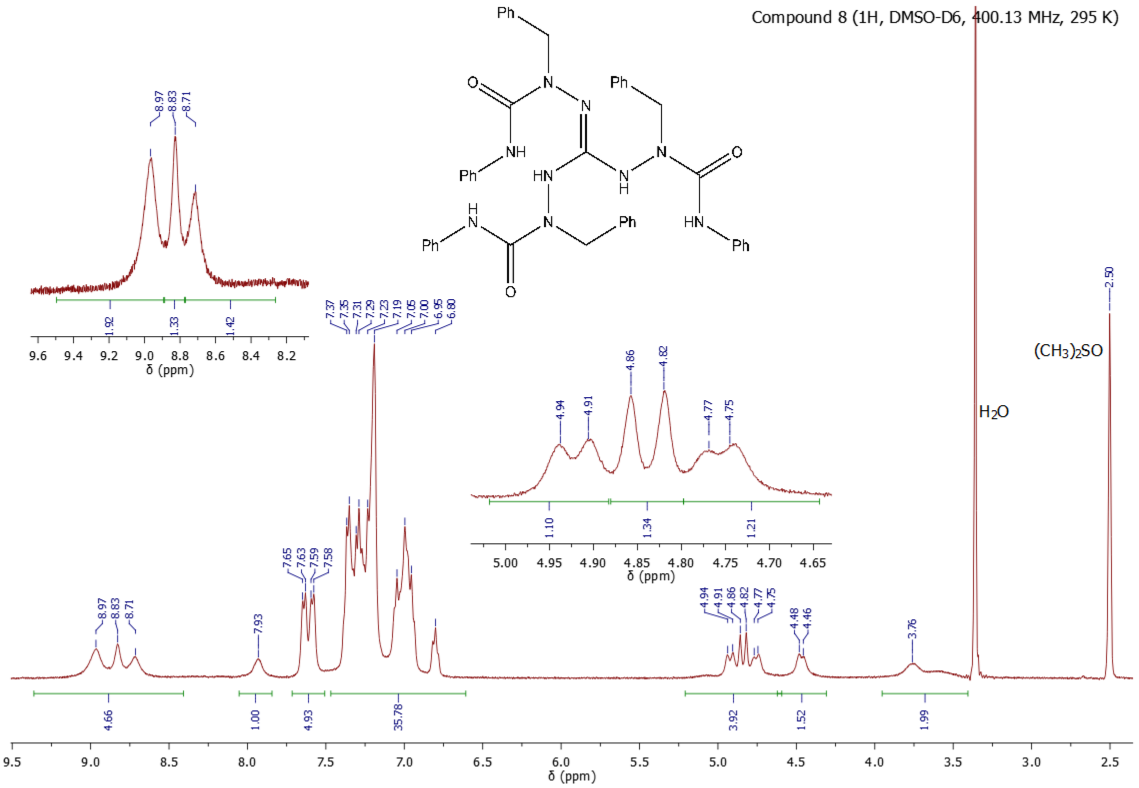

Figure S1. ^1H NMR spectrum of guanidine **8** ($[D_6]DMSO$, 400.13 MHz, at 295 K).

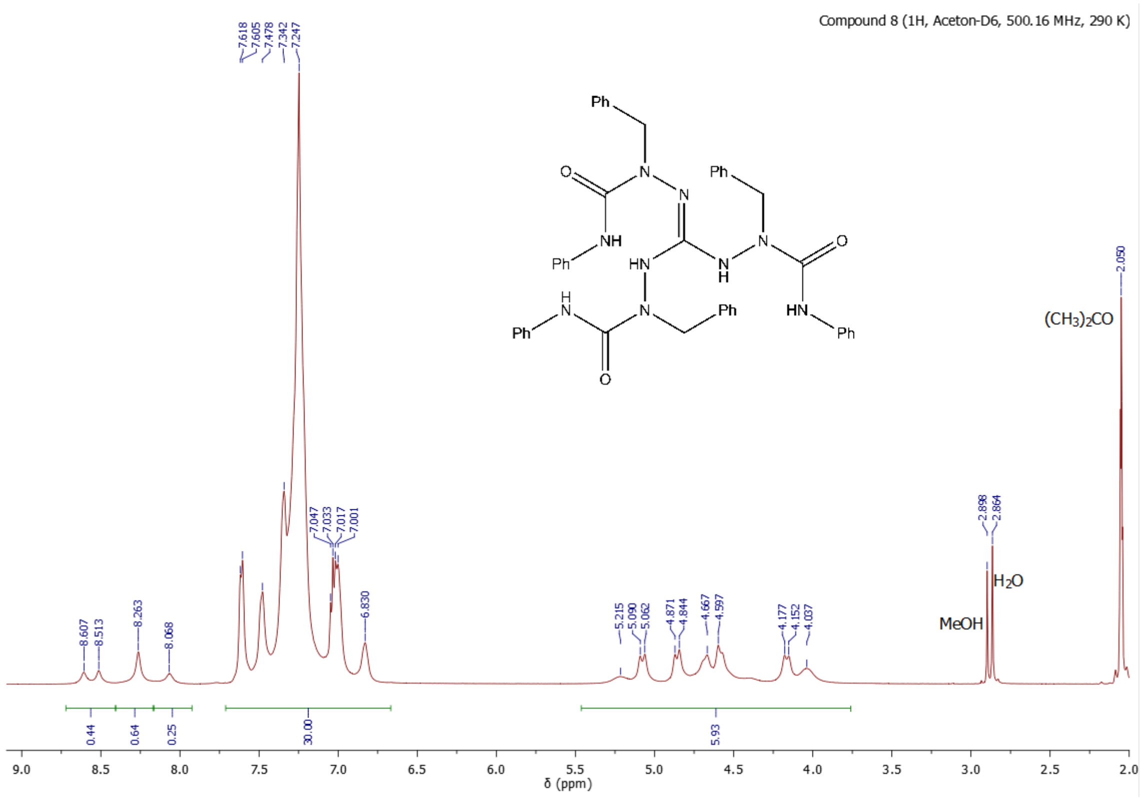

Figure S2. ^{1}H NMR spectrum of guanidine **8** ([D$_6$]acetone, 500.16 MHz at 290 K).

1,2-Bis(benzylamino)-3-[1-benzyl-3-(4-methylbenzenesulfonylureido)]guanidine (**9**): 1,2,3-Tris(benzylamino)guanidinium chloride (**3**) (4.00 g, 9.75 mmol) was dissolved in anhydrous $CHCl_3$ (130 mL) under an argon atmosphere, and (4-methylbenzenesulfonyl) isocyanate (2.3 mL, 15.1 mmol), in anhydrous $CHCl_3$ (20 mL) was added slowly. The reaction solution, which gradually assumed a rose color, was stirred at room temperature for 66 h. To this opaque solution silica gel (5.0 g) was added, and the solvent was evaporated at reduced pressure. The adsorbed solid was placed on a silica gel column and was eluted several times with ethyl acetate (3 × 60 mL) to remove several byproducts (TLC control). Subsequent elution with methanol/chloroform (1:1) furnished a dark yellow fraction, the solvent mixture of which was evaporated and replaced by methanol (30 mL). By addition of water the product could be precipitated as a white solid, which was filtered off, washed with

water and freeze-dried. A white powdry solid was obtained (1.68 g, 89 % yield), m. p. 176.2–177.2 °C. – IR (KBr): ν = ~ 3550–2700 (continuous absorption with maxima at 3279 (s), 3152 (s), 3062 (m), 3030 (m), 2945 (m), 2865 (m)), 1666 (s), 1616 (vs), 1496 (m), 1446 (m), 1354 (m), 1309 (s), 1259 (vs), 1145 (s), 1084 (m), 858 (s), 701 (s), 666 (m) cm^{-1}. – ^1H NMR ($(CD_3)_2SO$, 400.13 MHz): δ = 2.31 (s, 3 H, CH_3), 3.55/3.70 (two broad, unstructured and overlapping signals, 4 H, 2 × $NHCH_2Ph$), 4.22/4.62 (two broadened unstructured signals, 2 H, OCN-CH_AH_B), 5.30 (broadened s, 1 H, N*H*CH_2), 5.40 (broadened s, 1 H, N*H*CH_2), 7.12–7.28 (m, 15 H, H_{Ph}), 7.17/7.68 (AA'BB' spin system, 4 H, ^3J = 7.4 Hz, C_6H_4-4-CH_3), 8.42 (s, 1 H, NH), 8.76 (s, 1 H, NH), 9.02 (s, 1 H, NH) ppm. – ^{13}C NMR ($(CD_3)_2SO$, 125.76 MHz): δ = 20.91 (CH_3), 53.14 (OCN-CH_2), 54.38 (NCH_2), 54.59 (NCH_2); 127.05, 127.13, 127.34, 127.95, 128.17, 128.81, 129.00, 129.27, 136.74 (all CH_{Ar}); 136.90, 137.31, 139.26, 143.48 (all C_{Ar}); 157.2 and 160.33 (C=N and C=O) ppm. – MS (MALDI-TOF): *m/z* = 572.5 $[M + H]^+$, 375.5 [M – aryl-SO_2-NHCO + $H]^+$. – Anal. calcd. for $C_{30}H_{33}N_7O_3S$ (571.69): C, 63.03; H, 5.82; N, 17.15; S, 5.61; found: C, 63.16; H, 5.85; N, 17.23; S, 5.53.

3-Benzyl-3-{[1-benzyl-4-phenyl)-5-thioxo-1,2,4-triazol-3-yl]amino}-1-phenylthiourea (**10a**): 1,2,3-Tris(benzylamino)guanidinium chloride (**3**) (410 mg, 1.0 mmol) and phenyl isothiocyanate (477 µL, 4.0 mmol) were dissolved in $CHCl_3$ (20 mL), and the reaction mixture was heated at reflux for 3.5 h. The clear solution was concentrated to leave a colorless oil. This oil was dissolved in hot 2-propanol (20 mL), and the solution was cooled at 0 ^oC under vigorous stirring until a colorless precipitate formed, which was filtered off and washed with several portions of pentane. Another crop of the product was obtained from the mother liquor by repeating the described work-up procedure. The combined batches of product were dried at 30 ^oC/0.025

mbar, yielding a colorless powdry solid (298 mg, 57 % yield), m.p. 92.8–98.3 °C. – IR (KBr): ν = ~ 3300–2700 (series of broad, medium, strong and weak absorptions, maxima at 3245, 3030), 1596 (s), 1512 (s), 1445 (s), 1413 (m), 1328 (s), 1246 (m), 1182 (m), 1076 (m), 1036 (m), 1000 (m), 980 (w), 753 (m), 697 (s) cm^{-1}. – ^1H NMR ($(CD_3)_2SO$, 400.13 MHz): δ = 5.1–5.3 (broad signal in coalescence, 2 H, $PhCH_2NNH$), 5.28 (s, 2 H, $PhCH_2N_{ring}$), 7.19–7.50 (m, 20 H, H_{Ph}), 9.09 (s, 1 H, N-NH), 9.94 (s, 1 H, CS-NH) ppm. – ^{13}C NMR ($(CD_3)_2SO$, 100.62 MHz): δ = 51.14 (CH_2), 54.74 (CH_2); 125.52, 126.81, 127.43, 127.69, 127.97, 128.00, 128.04, 128.45, 128.49, 129.19, 129.25, 129.53 (all CH_{Ph}); 132.81, 135.85, 136.02, 139.94 (all C_{Ph}); 148.45 (C=N), 165.44 (N-CS-N), 181.74 (N-CS-NH) ppm. – HRMS (MALDI-TOF): m/z = 523.17333; $C_{29}H_{26}N_6S_2$ requires 5223.17331. – Anal. calcd. for $C_{29}H_{26}N_6S_2$ (522.69): C, 66.64; H, 5.01; N, 16.08; calcd. for $C_{29}H_{26}N_6S_2 \times 0.53\ H_2O$: C, 65.44; H, 5.12; N, 15.79; found: C, 65.45; H, 5.28; N, 15.97.

3-Benzyl-3-{[1-benzyl-4-(4-nitrophenyl)-5-thioxo-1,2,4-triazol-3-yl]amino}-1-(4-nitrophenyl)thiourea (**10b**): 1,2,3-Tris(benzylamino)guanidinium chloride (**3**) (744 mg, 1.81 mmol) and (*p*-nitrophenyl) isothiocyanate (1.31 g, 7.27 mmol) were dissolved in $CHCl_3$ (50 mL), and the reaction mixture was heated at reflux for 4 h. The clear yellow solution was concentrated to leave a yellow solid, which was first recrystallized from 2-propanol (from the mother liquor, by-product **11b** could be isolated, see below). The filtered and dried solid was triturated with a small volume of hot ethanol, the undissolved product was filtered off, washed with several portions of pentane and dried in vacuo at 40 ^oC/0.025 mbar. A yellow powdry solid was obtained (754 mg, 68 % yield), m. p. 132.5–133.6 °C. – IR (KBr): ν = 3296 (w), 1596 (m), 1528 (s), 1449 (m), 1411 (m), 1327 (s), 1247 (m), 1181 (m), 1109 (m), 999 (w), 851 (m), 701 (s) cm^{-1}. – ^1H NMR ($(CD_3)_2SO$, 500.16 MHz): δ = 5.0–5.5 (broad signal in

coalescence, 2 H, PhCH_2-N-NH), 5.33 (s, 2 H, PhCH_2-N_{ring}), 7.28–7.42 (m, 10 H, H_{benzyl}), 7.70/8.22 (AA'BB' system, ^3J = 8.6 Hz, 4 H, C_6H_4-4-NO_2), 7.70/8.34 (AA'BB' system, ^3J = 8.6 Hz, 4 H, C_6H_4-4-NO_2), 9.52 (s, 1 H, N-NH), 10.27 (s, 1 H, CS-NH) ppm. – ^{13}C NMR ($(CD_3)_2SO$, 125.76 MHz): δ = 51.75 (CH_2), 55.11 (CH_2); 124.09, 124.93, 126.05, 128.07, 128.30, 128.59, 128.98, 129.57, 130.75 (all CH_{Ar}); 135.88, 136.20, 138.74, 144.22, 146.35, 148.22 (all C_{Ar}); 148.40 (C=N), 165.83 (N-CS-N), 181.45 (N-CS-NH) ppm. – Anal. calcd. for $C_{29}H_{24}N_8O_4S_2$ (612.68): C, 56.85; H, 3.95; N, 18.29; S, 10.47; found: C, 56.90; H, 4.09; N, 18.22, S, 10.48.

3-Benzyl-1,6-bis(4-nitrophenyl)dithiourea (**11b**): Following the instructions of the synthesis of **10b** the mother liquor after the recrystallization with 2-propanol was evaporated. The residue was then triturated with hot acetone, leaving an insoluble yellow solid, which was filtered off and dried *in vacuo*. An orange powder was obtained (232 mg, 26 % yield), m. p. 151.1–156.0 °C. – IR (KBr): ν = 3448 (w), 2939 (w), 1615 (m), 1523 (br, s), 1331 (s), 1307 (s), 1254 (w), 1177 (w), 1106 (m), 1023 (w), 995 (m), 846 (m), 752 (w), 706 (w), 664 (w) cm^{-1}. – ^{1}H NMR ($(CD_3)_2SO$, 400.13 MHz): δ = 5.33 (s, 2 H, CH_2), 7.27/8.18 (AA'BB' spin system, ^3J = 9.0 Hz, 4 H, C_6H_4-4-NO_2), 7.30 (d, J = 7.4 Hz, 1 H, H_{Ph}), 7.38 (t, J = 7.4 Hz, 2 H, H_{Ph}), 7.45 (d, J = 7.4 Hz, 2 H, H_{Ph}), 7.61/8.13 (AA'BB' spin system, ^3J = 9.2 Hz, 4 H, C_6H_4-4-NO_2), 8.82 (s, 2 H, Aryl-NH-CS), 11.68 (s, 1 H, NNH) ppm. – ^{13}C NMR ($(CD_3)_2SO$, 100.62 MHz): δ = 52.06 (CH_2); 116.73, 121.43, 125.42, 125.63, 127.87, 128.23, 128.70 (all CH_{Ar}); 135.92, 140.82, 142.43, 144.89, 145.90 (all C_{Ar}); 154.91 (C=S), 155.93 (C=S) ppm. – Anal. calcd. for $C_{21}H_{18}N_6O_4S_2$ (482.54): C, 52.27; H, 3.76; N, 17.42; S, 13.29; found: C, 52.19; H, 3.66; N, 17.38; S, 13.40.

X-ray crystal structure determinations

Data collection was performed on an Oxford Diffraction instrument (SuperNova, Dual Source, Atlas CCD). Software for structure solution and refinement: SHELXS/L-97 [4, 5]; molecule plots: ORTEP-3 [6, 7].

Single crystals of **7a** were obtained by crystallization from anhydrous acetonitrile. They became opaque within ten seconds outside of the mother liquor. Therefore, they were immediately coated with a fluorinated oil (Fomblin® YR-1800), a suitable crystal was chosen, mounted on the crystal holder of the diffractometer, and cooled to the temperature of the data collection. In the structure refinement procedure, all hydrogen atom positions were allowed to refine freely, except for the hydrogen atoms of the three acetonitrile molecules, which were placed in geometrically calculated positions and treated as riding on the adjacent carbon atom.

Single crystals of **8** and **10b** were obtained by diffusion of pentane from the vapor phase into solutions of the two compounds in ethyl acetate. Hydrogen atoms were included in the structure refinement procedure either in geometrically calculated positions or in positions taken from a ΔF map (all N−H hydrogen atoms) and treated as riding on their bond neighbors. In the unit cell of **8**, a region of residual electron density was observed, which most likely arose from highly disordered pentane molecules and could not be resolved. Its contribution to the reflection data set was removed using the SQUEEZE function of PLATON [8]; this led to a residual electron density of 0.28 e $Å^{-3}$.

Further details are provided in Table 1. CCDC 1001264 (**7a**), 1001265 (**8**) and 1001266 (**10b**) contain the supplementary crystallographic data for this paper. These data can be obtained free of charge from The Cambridge Crystallographic Data Centre *via* www.ccdc.cam.ac.uk/data_request/cif.

Table S1: Crystal structure data for **7a**, **8**, and **10b**.

	**7a**	**8**	**10b**
Formula	$C_{43}H_{42}ClN_9O_3 \times$ 3 (CH_3CN)	$C_{43}H_{41}N_9O_3$	$C_{29}H_{24}N_8O_4S_2$
M_r	768.31 + 123.16	731.85	612.68
Cryst. size, mm^3	0.24 × 0.20 × 0.16	0.27 × 0.15 × 0.12	0.36 × 0.14 × 0.08
Crystal system	triclinic	triclinic	orthorhombic
Space group	*P*-1	*P*-1	*Pbcn*
a, Å	12.3038(4)	12.1587(7)	23.9765(2)
b, Å	14.5135(6)	13.7650(8)	10.11359(7)
c, Å	15.6425(6)	14.9306(10)	23.5681(2)
α, deg	63.955(4)	117.387(6)	90
β, deg	76.030(3)	107.798(5)	90
γ, deg	81.703(3)	95.577(5)	90
V, $Å^3$	2433.1(2)	2027.3(2)	5715.01(8)
Z	2	2	8
D_{calcd}, g cm^{-3}	1.22	1.20	1.42
$\mu(MoK_\alpha)$, mm^{-1}	1.12	0.63	2.12
F(000), e	940	772	2544
Radiation	CuK_α	CuK_α	CuK_α
Temperature, K	190(2)	190(2)	190(2)
hkl range	$-15 \le h \le +14$, $-18 \le k \le 17$, $-19 \le l \le +19$	$-15 \le h \le +14$, $-17 \le k \le 11$, $-17 \le l \le +18$	$-29 \le h \le +20$, $-8 \le k \le 12$, $-26 \le l \le +18$
θ range, deg	3.21 – 74.14	3.63 – 74.01	3.75 – 73.73
Refl. measured	34834	14310	10102
Refl. unique	9564	7954	5343
R_{int}	0.0350	0.0288	0.0267
Param. refined / restraints	757 / 0	516 / 0	396 / 0
$R(F)/wR(F^2)^a$ ($I \ge 2\sigma(I)$)	0.0465/0.1228	0.0477/0.1306	0.0419/0.1085
$R(F)/wR(F^2)^a$ (all reflexions)	0.0623/0.1275	0.0609/0.1416	0.0511/0.1170
$GoF\ (F^2)^a$	1.242	1.041	1.030
$\Delta\rho_{fin}$ (max/min), e $Å^{-3}$	0.24, -0.26	0.28, -0.19	0.55, -0.41

[a] $R(F) = \Sigma||F_o| - |F_c| / \Sigma|F_o|$; $wR(F^2) = [\Sigma(w(F_o^2 - F_c^2)^2) / \Sigma w(F_o^2)^2]^{1/2}$; $GoF = [\Sigma w(F_o^2 - F_c^2)^2 / (n_{obs} - n_{param})]^{1/2}$.

Table S2: Bond distances [Å] and angles [^{o}] for **7a**.

O(1)-C(2)	1.228(3)	C(24)-C(25)	1.387(4)
O(2)-C(16)	1.216(3)	C(25)-C(26)	1.381(4)
O(3)-C(30)	1.215(3)	C(26)-C(27)	1.356(6)
N(1)-C(1)	1.330(3)	C(27)-C(28)	1.370(6)
N(1)-N(2)	1.397(3)	C(28)-C(29)	1.403(5)
N(2)-C(2)	1.406(3)	C(31)-C(32)	1.384(4)
N(2)-C(9)	1.484(3)	C(31)-C(36)	1.388(4)
N(3)-C(2)	1.341(3)	C(32)-C(33)	1.386(4)
N(3)-C(3)	1.425(3)	C(33)-C(34)	1.376(5)
N(4)-C(1)	1.333(3)	C(34)-C(35)	1.368(6)
N(4)-N(5)	1.388(3)	C(35)-C(36)	1.395(5)
N(5)-C(16)	1.392(3)	C(37)-C(38)	1.493(4)
N(5)-C(23)	1.470(3)	C(38)-C(43)	1.383(5)
N(6)-C(16)	1.355(3)	C(38)-C(39)	1.384(4)
N(6)-C(17)	1.416(3)	C(39)-C(40)	1.386(6)
N(7)-C(1)	1.332(3)	C(40)-C(41)	1.372(7)
N(7)-N(8)	1.401(3)	C(41)-C(42)	1.356(7)
N(8)-C(30)	1.424(3)	C(42)-C(43)	1.386(6)
N(8)-C(37)	1.477(4)	N(10)-C(44)	1.123(4)
N(9)-C(30)	1.344(4)	C(44)-C(45)	1.457(4)
N(9)-C(31)	1.421(3)	N(11)-C(46)	1.128(4)
C(3)-C(8)	1.384(4)	C(46)-C(47)	1.454(5)
C(3)-C(4)	1.385(4)	N(12)-C(48)	1.141(7)
C(4)-C(5)	1.382(4)	C(48)-C(49)	1.443(7)
C(5)-C(6)	1.377(4)		
C(6)-C(7)	1.385(5)	C(1)-N(1)-N(2)	120.9(2)
C(7)-C(8)	1.384(4)	N(1)-N(2)-C(2)	116.05(19)
C(9)-C(10)	1.503(4)	N(1)-N(2)-C(9)	115.2(2)
C(10)-C(11)	1.384(4)	C(2)-N(2)-C(9)	117.9(2)
C(10)-C(15)	1.389(4)	C(2)-N(3)-C(3)	122.6(2)
C(11)-C(12)	1.382(5)	C(1)-N(4)-N(5)	118.7(2)
C(12)-C(13)	1.371(6)	N(4)-N(5)-C(16)	121.3(2)
C(13)-C(14)	1.370(6)	N(4)-N(5)-C(23)	117.3(2)
C(14)-C(15)	1.384(5)	C(16)-N(5)-C(23)	117.3(2)
C(17)-C(18)	1.389(4)	C(16)-N(6)-C(17)	125.3(2)
C(17)-C(22)	1.392(4)	C(1)-N(7)-N(8)	120.0(2)
C(18)-C(19)	1.391(5)	N(7)-N(8)-C(30)	117.5(2)
C(19)-C(20)	1.369(6)	N(7)-N(8)-C(37)	112.5(2)
C(20)-C(21)	1.374(6)	C(30)-N(8)-C(37)	116.6(2)
C(21)-C(22)	1.382(4)	C(30)-N(9)-C(31)	124.8(2)
C(23)-C(24)	1.508(4)	N(1)-C(1)-N(7)	120.7(2)
C(24)-C(29)	1.378(4)	N(1)-C(1)-N(4)	119.0(2)

N(7)-C(1)-N(4)	120.3(2)	N(5)-C(23)-C(24)	115.4(2)
O(1)-C(2)-N(3)	124.9(2)	C(29)-C(24)-C(25)	118.8(3)
O(1)-C(2)-N(2)	119.3(2)	C(29)-C(24)-C(23)	118.4(3)
N(3)-C(2)-N(2)	115.7(2)	C(25)-C(24)-C(23)	122.8(3)
C(8)-C(3)-C(4)	120.0(3)	C(26)-C(25)-C(24)	120.8(3)
C(8)-C(3)-N(3)	118.8(2)	C(27)-C(26)-C(25)	120.4(4)
C(4)-C(3)-N(3)	121.1(2)	C(26)-C(27)-C(28)	119.9(4)
C(5)-C(4)-C(3)	119.7(3)	C(27)-C(28)-C(29)	120.5(4)
C(6)-C(5)-C(4)	120.6(3)	C(24)-C(29)-C(28)	119.5(4)
C(5)-C(6)-C(7)	119.7(3)	O(3)-C(30)-N(9)	125.7(3)
C(8)-C(7)-C(6)	120.1(3)	O(3)-C(30)-N(8)	118.8(3)
C(7)-C(8)-C(3)	119.9(3)	N(9)-C(30)-N(8)	115.4(2)
N(2)-C(9)-C(10)	114.4(2)	C(32)-C(31)-C(36)	119.5(3)
C(11)-C(10)-C(15)	118.7(3)	C(32)-C(31)-N(9)	117.5(3)
C(11)-C(10)-C(9)	119.9(3)	C(36)-C(31)-N(9)	123.0(3)
C(15)-C(10)-C(9)	121.4(3)	C(31)-C(32)-C(33)	120.5(3)
C(12)-C(11)-C(10)	120.8(3)	C(34)-C(33)-C(32)	120.2(4)
C(13)-C(12)-C(11)	119.8(4)	C(35)-C(34)-C(33)	119.4(3)
C(14)-C(13)-C(12)	120.3(4)	C(34)-C(35)-C(36)	121.5(3)
C(13)-C(14)-C(15)	120.3(4)	C(31)-C(36)-C(35)	118.8(3)
C(14)-C(15)-C(10)	120.1(3)	N(8)-C(37)-C(38)	113.7(2)
O(2)-C(16)-N(6)	125.3(2)	C(43)-C(38)-C(39)	117.9(3)
O(2)-C(16)-N(5)	118.5(2)	C(43)-C(38)-C(37)	120.5(3)
N(6)-C(16)-N(5)	116.1(2)	C(39)-C(38)-C(37)	121.5(3)
C(18)-C(17)-C(22)	119.4(3)	C(38)-C(39)-C(40)	120.9(4)
C(18)-C(17)-N(6)	124.2(3)	C(41)-C(40)-C(39)	119.8(4)
C(22)-C(17)-N(6)	116.4(3)	C(42)-C(41)-C(40)	120.3(4)
C(17)-C(18)-C(19)	119.2(3)	C(41)-C(42)-C(43)	120.1(5)
C(20)-C(19)-C(18)	121.3(4)	C(38)-C(43)-C(42)	121.0(4)
C(19)-C(20)-C(21)	119.3(3)	N(10)-C(44)-C(45)	179.8(3)
C(20)-C(21)-C(22)	120.8(4)	N(11)-C(46)-C(47)	178.2(5)
C(21)-C(22)-C(17)	119.9(3)	N(12)-C(48)-C(49)	177.9(8)

Table S3: Bond distances (Å) and angles (^{o}) for **8**.

O(1)-C(2)	1.2351(17)	C(25)-C(26)	1.383(3)
O(2)-C(16)	1.2348(16)	C(26)-C(27)	1.361(4)
O(3)-C(30)	1.2167(19)	C(27)-C(28)	1.365(4)
N(1)-C(1)	1.3049(18)	C(28)-C(29)	1.387(3)
N(1)-N(4)	1.4329(16)	C(31)-C(36)	1.376(3)
N(2)-C(1)	1.3821(18)	C(31)-C(32)	1.384(2)
N(2)-N(6)	1.4073(15)	C(32)-C(33)	1.386(3)
N(3)-C(1)	1.3512(17)	C(33)-C(34)	1.360(3)
N(3)-N(8)	1.4070(17)	C(34)-C(35)	1.376(3)
N(4)-C(2)	1.366(2)	C(35)-C(36)	1.370(3)
N(4)-C(9)	1.4652(19)	C(37)-C(38)	1.503(2)
N(5)-C(2)	1.362(2)	C(38)-C(39)	1.375(3)
N(5)-C(3)	1.406(2)	C(38)-C(43)	1.384(3)
N(6)-C(16)	1.3818(18)	C(39)-C(40)	1.390(3)
N(6)-C(23)	1.4603(17)	C(40)-C(41)	1.372(3)
N(7)-C(16)	1.3633(18)	C(41)-C(42)	1.365(3)
N(7)-C(17)	1.4271(19)	C(42)-C(43)	1.386(3)
N(8)-C(30)	1.423(2)		
N(8)-C(37)	1.4746(19)	C(1)-N(1)-N(4)	111.70(11)
N(9)-C(30)	1.352(2)	C(1)-N(2)-N(6)	116.40(11)
N(9)-C(31)	1.406(2)	C(1)-N(3)-N(8)	121.36(12)
C(3)-C(8)	1.381(2)	C(2)-N(4)-N(1)	118.13(11)
C(3)-C(4)	1.391(3)	C(2)-N(4)-C(9)	120.38(12)
C(4)-C(5)	1.386(3)	N(1)-N(4)-C(9)	117.33(12)
C(5)-C(6)	1.375(4)	C(2)-N(5)-C(3)	126.96(14)
C(6)-C(7)	1.369(4)	C(16)-N(6)-N(2)	115.22(11)
C(7)-C(8)	1.385(3)	C(16)-N(6)-C(23)	125.92(11)
C(9)-C(10)	1.506(2)	N(2)-N(6)-C(23)	113.60(11)
C(10)-C(15)	1.377(3)	C(16)-N(7)-C(17)	120.85(12)
C(10)-C(11)	1.380(3)	N(3)-N(8)-C(30)	115.52(12)
C(11)-C(12)	1.386(3)	N(3)-N(8)-C(37)	113.36(12)
C(12)-C(13)	1.374(4)	C(30)-N(8)-C(37)	113.59(12)
C(13)-C(14)	1.357(4)	C(30)-N(9)-C(31)	127.43(14)
C(14)-C(15)	1.386(3)	N(1)-C(1)-N(3)	124.98(13)
C(17)-C(22)	1.384(2)	N(1)-C(1)-N(2)	118.83(12)
C(17)-C(18)	1.386(2)	N(3)-C(1)-N(2)	116.18(12)
C(18)-C(19)	1.381(3)	O(1)-C(2)-N(5)	123.50(15)
C(19)-C(20)	1.385(3)	O(1)-C(2)-N(4)	121.79(14)
C(20)-C(21)	1.373(3)	N(5)-C(2)-N(4)	114.67(13)
C(21)-C(22)	1.386(3)	C(8)-C(3)-C(4)	118.95(17)
C(23)-C(24)	1.498(2)	C(8)-C(3)-N(5)	123.79(17)
C(24)-C(29)	1.377(3)	C(4)-C(3)-N(5)	117.09(15)
C(24)-C(25)	1.382(3)	C(5)-C(4)-C(3)	120.6(2)

C(6)-C(5)-C(4)	120.0(2)	C(25)-C(24)-C(23)	120.27(16)
C(7)-C(6)-C(5)	119.3(2)	C(24)-C(25)-C(26)	120.8(2)
C(6)-C(7)-C(8)	121.5(2)	C(27)-C(26)-C(25)	119.7(2)
C(3)-C(8)-C(7)	119.6(2)	C(26)-C(27)-C(28)	120.4(2)
N(4)-C(9)-C(10)	114.60(12)	C(27)-C(28)-C(29)	120.3(3)
C(15)-C(10)-C(11)	118.08(18)	C(24)-C(29)-C(28)	120.0(2)
C(15)-C(10)-C(9)	120.61(17)	O(3)-C(30)-N(9)	125.49(15)
C(11)-C(10)-C(9)	121.28(18)	O(3)-C(30)-N(8)	120.02(15)
C(10)-C(11)-C(12)	120.5(2)	N(9)-C(30)-N(8)	114.40(13)
C(13)-C(12)-C(11)	120.5(2)	C(36)-C(31)-C(32)	118.23(17)
C(14)-C(13)-C(12)	119.4(2)	C(36)-C(31)-N(9)	117.07(15)
C(13)-C(14)-C(15)	120.4(2)	C(32)-C(31)-N(9)	124.68(17)
C(10)-C(15)-C(14)	121.1(2)	C(31)-C(32)-C(33)	119.85(19)
O(2)-C(16)-N(7)	122.51(13)	C(34)-C(33)-C(32)	121.32(19)
O(2)-C(16)-N(6)	119.58(12)	C(33)-C(34)-C(35)	118.8(2)
N(7)-C(16)-N(6)	117.85(12)	C(36)-C(35)-C(34)	120.4(2)
C(22)-C(17)-C(18)	119.49(15)	C(35)-C(36)-C(31)	121.35(19)
C(22)-C(17)-N(7)	119.75(14)	N(8)-C(37)-C(38)	113.64(13)
C(18)-C(17)-N(7)	120.74(14)	C(39)-C(38)-C(43)	118.23(17)
C(19)-C(18)-C(17)	119.93(18)	C(39)-C(38)-C(37)	121.32(16)
C(18)-C(19)-C(20)	120.58(19)	C(43)-C(38)-C(37)	120.29(16)
C(20)-C(21)-C(22)	120.5(2)	C(38)-C(39)-C(40)	120.79(19)
C(21)-C(20)-C(19)	119.39(19)	C(41)-C(40)-C(39)	120.10(19)
C(17)-C(22)-C(21)	120.10(17)	C(42)-C(41)-C(40)	119.80(19)
N(6)-C(23)-C(24)	114.01(12)	C(41)-C(42)-C(43)	120.1(2)
C(29)-C(24)-C(25)	118.83(17)	C(38)-C(43)-C(42)	120.93(19)
C(29)-C(24)-C(23)	120.89(16)		

Table S4: Selected torsion angles in the solid-state structures of **7a** and **8** (^{o}).

Salt **7a**		Guanidine **8**	
C1–N4–N5–C16	115.3(3)	C1–N1–N4–C2	110.9(1)
C1–N1–N2–C2	127.3(2)	C1–N2–N6–C16	65.6(2)
C1–N7–N8–C30	118.9(3)	C1–N3–N8–C30	113.6(2)
C1–N1–N2–C9	-89.1(3)	C1–N1–N4–C9	-92.0(1)

C1–N4–N5–C23	-88.0(3)	C1–N2–N6–C23	-138.4(1)
C1–N7–N8–C37	-101.5(3)	C1–N3–N8–C37	-112.9(2)
N1–N2–C2–O1	153.0(2)	N1–N4–C2–O1	161.8(1)
N4–N5–C16–O2	165.8(2)	N2–N6–C16–O2	-13.3(2)
N7–N8–C30–O3	158.7(2)	N3–N8–C30–O3	146.3(2)
		N1–C1–N2–N6	35.9(2)
		N1–C1–N3–N8	-178.0(1)

Table S5: Bond distances (Å) and angles (°) for **10b**.

S(1)-C(2)	1.664(2)
S(2)-C(23)	1.672(2)
N(1)-C(1)	1.388(3)
N(1)-N(2)	1.413(2)
N(2)-C(23)	1.377(2)
N(2)-C(3)	1.484(2)
N(3)-C(1)	1.298(2)
N(3)-N(4)	1.381(3)
N(4)-C(2)	1.346(3)
N(4)-C(10)	1.466(3)
N(5)-C(1)	1.374(2)
N(5)-C(2)	1.382(3)
N(5)-C(17)	1.443(2)
N(6)-O(2)	1.223(3)
N(6)-O(1)	1.227(3)
N(6)-C(20)	1.471(3)
N(7)-C(23)	1.353(3)
N(7)-C(24)	1.412(2)
N(8)-O(4)	1.213(3)
N(8)-O(3)	1.217(3)
N(8)-C(27)	1.466(3)
C(3)-C(4)	1.516(3)
C(4)-C(9)	1.385(3)
C(4)-C(5)	1.396(3)
C(5)-C(6)	1.391(3)
C(6)-C(7)	1.381(4)
C(7)-C(8)	1.389(4)
C(8)-C(9)	1.390(3)
C(10)-C(11)	1.497(3)
C(11)-C(16)	1.372(3)
C(11)-C(12)	1.387(3)
C(12)-C(13)	1.361(4)
C(13)-C(14)	1.354(5)
C(14)-C(15)	1.349(5)
C(15)-C(16)	1.436(5)
C(17)-C(18)	1.380(3)
C(17)-C(22)	1.385(3)
C(18)-C(19)	1.388(3)
C(19)-C(20)	1.379(3)
C(20)-C(21)	1.380(3)
C(21)-C(22)	1.392(3)
C(24)-C(25)	1.394(3)
C(24)-C(29)	1.396(3)
C(25)-C(26)	1.377(3)
C(26)-C(27)	1.380(3)
C(27)-C(28)	1.381(3)
C(28)-C(29)	1.386(3)

C(1)-N(1)-N(2)	116.18(14)
C(23)-N(2)-N(1)	116.79(15)
C(23)-N(2)-C(3)	122.59(17)
N(1)-N(2)-C(3)	115.80(14)

C(1)-N(3)-N(4)	103.77(16)
C(2)-N(4)-N(3)	113.08(16)
C(2)-N(4)-C(10)	127.54(19)
N(3)-N(4)-C(10)	119.04(17)
C(1)-N(5)-C(2)	107.12(15)
C(1)-N(5)-C(17)	128.02(16)
C(2)-N(5)-C(17)	124.75(16)
O(2)-N(6)-O(1)	124.10(18)
O(2)-N(6)-C(20)	118.16(19)
O(1)-N(6)-C(20)	117.74(18)
C(23)-N(7)-C(24)	129.80(18)
O(4)-N(8)-O(3)	123.1(2)
O(4)-N(8)-C(27)	118.2(2)
O(3)-N(8)-C(27)	118.7(2)
N(3)-C(1)-N(5)	112.21(17)
N(3)-C(1)-N(1)	122.95(17)
N(5)-C(1)-N(1)	124.61(16)
N(4)-C(2)-N(5)	103.80(17)
N(4)-C(2)-S(1)	128.73(16)
N(5)-C(2)-S(1)	127.46(15)
N(2)-C(3)-C(4)	113.91(16)
C(9)-C(4)-C(5)	119.29(19)
C(9)-C(4)-C(3)	121.17(18)
C(5)-C(4)-C(3)	119.5(2)
C(6)-C(5)-C(4)	120.0(2)
C(7)-C(6)-C(5)	120.2(2)
C(6)-C(7)-C(8)	120.1(2)
C(7)-C(8)-C(9)	119.7(2)
C(4)-C(9)-C(8)	120.6(2)
N(4)-C(10)-C(11)	114.06(17)
C(16)-C(11)-C(12)	118.3(2)
C(16)-C(11)-C(10)	122.1(2)
C(12)-C(11)-C(10)	119.4(2)
C(13)-C(12)-C(11)	121.9(3)
C(14)-C(13)-C(12)	119.4(3)
C(15)-C(14)-C(13)	121.9(3)
C(14)-C(15)-C(16)	118.7(3)
C(11)-C(16)-C(15)	119.5(3)
C(18)-C(17)-C(22)	122.05(17)
C(18)-C(17)-N(5)	118.41(16)
C(22)-C(17)-N(5)	119.54(16)
C(17)-C(18)-C(19)	119.49(18)
C(20)-C(19)-C(18)	118.11(18)
C(19)-C(20)-C(21)	123.02(18)
C(19)-C(20)-N(6)	117.86(18)
C(21)-C(20)-N(6)	119.12(19)
C(20)-C(21)-C(22)	118.57(19)
C(17)-C(22)-C(21)	118.72(18)
N(7)-C(23)-N(2)	114.70(17)
N(7)-C(23)-S(2)	124.10(15)
N(2)-C(23)-S(2)	121.20(14)
C(25)-C(24)-C(29)	119.40(18)
C(25)-C(24)-N(7)	116.74(17)
C(29)-C(24)-N(7)	123.69(17)
C(26)-C(25)-C(24)	121.23(18)
C(25)-C(26)-C(27)	118.22(18)
C(26)-C(27)-C(28)	122.16(19)
C(26)-C(27)-N(8)	118.5(2)
C(28)-C(27)-N(8)	119.4(2)
C(27)-C(28)-C(29)	119.27(19)
C(28)-C(29)-C(24)	119.65(19)

References

1. Weiss, S.; Krommer, H. (SKW Trostberg AG), DE3341645 (A1), **1985**; Chem. Abstr. **1986**, *104*, 206730.
2. Bucher, N.; Szabo, J.; Oppel, I. M.; Maas, G. *Z. Naturforsch.* **2012**, 67b, 631–642.
3. Busch-Petersen, J. U.S. Patent 2007/0,249,672, October 25, **2007**.
4. Sheldrick, G. M., SHELXS/L-97, Program for the Solution and Refinement of Crystal Structures from Diffraction Data, University of Göttingen, Göttingen (Germany), **1997**.
5. Sheldrick, G. M. *Acta Crystallogr.* **2008**, *A64*, 112–122.
6. Johnson, C. K.; Burnett, M. N., ORTEP-3 (version 1.0.2), Rep. ORNL-6895, Oak Ridge National Laboratory, Oak Ridge, TN (USA), **1996**.
7. Farrugia, L. J., ORTEP, Windows version, University of Glasgow, Glasgow (Scotland), **1997–2008**.
8. (a) Spek, A. L. *Acta Crystallogr., Sect A* **1990**, *46*, C34. (b) Spek, A. L., PLATON, A Multipurpose Crystallographic Tool, Utrecht University, Utrecht, The Netherlands, **1998**.
